# Advancements in Polymeric Nanocarriers to Mediate Targeted Therapy against Triple-Negative Breast Cancer

**DOI:** 10.3390/pharmaceutics14112432

**Published:** 2022-11-10

**Authors:** Mahak Fatima, Afsana Sheikh, Mohammed A. S. Abourehab, Prashant Kesharwani

**Affiliations:** 1 Department of Pharmaceutics, School of Pharmaceutical Education and Research, Jamia Hamdard, New Delhi 110062, India; 2 Department of Pharmaceutics, College of Pharmacy, Umm Al-Qura University, Makkah 21955, Saudi Arabia; 3 Center for Transdisciplinary Research, Department of Pharmacology, Saveetha Dental College, Saveetha Institute of Medical and Technical Science, Chennai 602105, India

**Keywords:** triple-negative breast cancer, drug-delivery system, polymeric nanoparticles, nanotechnology, immunotherapy, cancer-stem cells

## Abstract

Triple-negative breast cancer (TNBC) is a destructive disease with a poor prognosis, low survival rate and high rate of metastasis. It comprises 15% of total breast cancers and is marked by deficiency of three important receptor expressions, i.e., progesterone, estrogen, and human epidermal growth factor receptors. This absence of receptors is the foremost cause of current TNBC therapy failure, resulting in poor therapeutic response in patients. Polymeric nanoparticles are gaining much popularity for transporting chemotherapeutics, genes, and small-interfering RNAs. Due to their exclusive properties such as great stability, easy surface modification, stimuli-responsive and controlled drug release, ability to condense more than one therapeutic moiety inside, tumor-specific delivery of payload, enhanced permeation and retention effect, present them as ideal nanocarriers for increasing efficacy, bioavailability and reducing the toxicity of therapeutic agents. They can even be used as theragnostic agents for the diagnosis of TNBC along with its treatment. In this review, we discuss the limitations of already existing TNBC therapies and highlight the novel approach to designing and the functionalization of polymeric nanocarriers for the effective treatment of TNBC.

## 1. Introduction

Oncology is a growing field in the healthcare sector, as cancer is the second most common reason for mortality round the globe. With the highest prevalence of breast cancer with 2.26 million cases, followed by 2.21 million cases of lung cancer, and 1.9 million cases of colorectal and 1.4 million cases of prostate cancer, globally [1]. According to health organization reports, 10 million people died due to cancer in 2020 alone [2]. As the highest prevailing cancer is breast cancer (BC), it is the leading cause of mortality among females and is considered a major reason of cancer burden [3,4,5,6].

There are certain endocrines present in the human mammary gland. Progesterone (PR), human epidermal growth factor (HER-2), and estrogen (ER) are major endocrines [7]. Depending on the presence or absence of certain receptors for these endocrines, breast cancer is divided into three divisions: (1) HER-2 positive BC (2) hormone receptor-positive BC (PR+ and ER+) and (3) triple-negative BC (TNBC) [8]. Among all three, TNBC is the most aggressive form of BC as it lacks all three receptors, i.e., there is an absence of PR, ER and HER-2 receptors in this type of cancer. TNBC does not respond to available endocrine therapies, as these therapies include PR, ER and HER-2 receptor targeting. Various other therapies are available for treating breast cancer that include immunotherapy, endocrine therapies, radiotherapy, and chemotherapy [9,10,11,12,13,14,15,16,17]. Thus, oncologists have been forced to investigate and utilize chemotherapeutic agents [18,19,20,21,22,23,24,25]; however, due to the associated complications, the treatment regimen lengthens and the rate of survival decreases. The complications include high toxicity, low bioavailability, emerging resistance and low cellular uptake. TNBC has the highest rate of metastatic recurrences, the lowest overall rate of survival which are being accountable for 15% of all invasive BC [12,25,26,27,28,29,30]. Along with this, there are numerous cases of distant recurrences reported along with a gradual development of chemotherapeutic resistance developed against its sole existing treatment regimen chiefly based on taxanes and anthracyclines [31]. Therefore, treating TNBC is a great obstacle that cannot be mitigated by existing therapies. Hence, advancement in therapy regimens for TNBC is an urgent need.

Over the decades, significant advances are made in the field of cancer therapy [32,33,34,35,36]. However, attaining a complete cure still seems to be an implausible dream. This is because of the different complications associated with cancer cells. Several anti-cancer drugs such as paclitaxel (PTX), cisplatin, etoposide, docetaxel (DCL), and camptothecin have received a green signal from the US-FDA (Food and Drug Administration). These drugs are available as monotherapies as well as combinatorial therapies to check the progress of cancer. Nonetheless, almost all of these clinically used drugs belong to class III or IV of the BCS (biopharmaceutical classification system). So they possess low aqueous solubility, poor dissolution and permeability, thus low bioavailability [3]. Due to their poor solubility and permeability, they are incapable of penetrating the tumor cells and attaining the desired concentration inside. This issue is compensated for by using a high dose of drugs and multi-drug regimens, which bring uninvited side effects due to the non-specific targeting of healthy cells by such drugs, all over the body. In addition to this, multi-drug resistance (MDR) and insufficient early diagnostic methodologies are also major hindrances in cancer treatment [37]. To subdivide this heterogeneous cancer, mRNA signatures, protein expressions, as well as genomic variations are considered [38]. The therapeutic efficiency of small molecules such as genes, plasmids, siRNA (small interfering RNA) that are highly efficacious in prohibiting cancer progression in various cancers are curbed by their in vivo instability, unspecific cell targeting, low immunogenicity, large size, negative surface charge and poor aqueous solubility [25,28,39,40,41,42].

Nanotechnology approaches may be a providential way for battling cancer. It allows for specific tumor cell targeting, improve bio-distribution, bio-availability, enhanced permeation and retention (EPR) effect, modifiable surface, pH-dependent drug release and reduced toxicity [43,44,45,46]. The size of the targeting molecule has a crucial role in tumor treatment. The size of the particle should be small enough that it can easily penetrate the tumor cells and large enough to be able to retain it inside the tumor microenvironment (TME). Hence, the nanodrug transport system should be designed with a modifiable structure to attain the desired results [47,48,49,50,51,52,53,54].

Polymeric nanodrug delivery systems are gaining immense attention because of their biodegradable and biocompatible nature. The drug can be physically or chemically entrapped inside these nanoparticles (NPs) and their surface can be modified to deliver the drug specifically into tumor cells, overcoming the challenge of non-specific drug delivery. These NPs are being used as powerful tools for the intelligent treatment of cancer cells. This delivery system imparts improved stability, and solubility of the loaded drug, and prolongs the blood circulation and retention time of the NPs at the tumor site [55,56,57,58]. The EPR effect of polymeric NPs leads to high intra-tumoral deposition of the drug as an outcome of the malformed permeable capillaries and insignificant lymphoid drainage of the cancer cells [59,60]. NPs within the size range of 8 to 100 nanometers (nm) can target cancer cells via the EPR effect (passive targeting) and thus achieve an enhanced drug dispersal inside the tumor, contributing to the overall improved therapeutic efficacy of the encapsulated drug [61,62,63].

Polymeric NPs showed immense potential in pre-clinical studies as a diagnostic agent as well as an effective carrier for small-sized hydrophobic drugs for cancer therapy. Polymeric NPs designed using amphiphilic block co-polymers are of a diversified nature. Researchers explored and formulated different polymeric combinations, each of them having a diverse function and modifiable chemistry [64]. Amphiphilic block co-polymers offer stimuli-responsive characteristics that cater the drug delivery into the TME [65]. pH, temperature, light, redox state, hypoxia, and reactive oxygen species (ROS) are among the different stimuli that are exploited for attaining tumor-specificity. Herein, we discuss some of the stimuli-responsive polymeric NPs, their detailed molecular mechanism, and strategies behind stimuli-responsive drug release from nanocarriers.

## 2. Stimuli-Responsive Polymeric Nanoparticles

The modifiable chemistry of polymers and co-polymers allows for post-polymerization modification via chemical processes, such as using a covalent-coupling process that offers many opportunities to polish up the polymers at the sub-molecular and molecular levels [65]. Alcohols, carboxylic acids, and amines are functional groups present in polymers and co-polymers that are generally taken advantage of for chemically modifying polymers [66]. Such stimuli-responsive NPs have attracted great interest to deliver payloads to specific tumor sites under desired conditions. Several stimuli, both extrinsic (light, ultrasound, temperature, and magnetic) as well as intrinsic (pH, hypoxia, ROS, enzyme, and redox) to the biological structure, have been exploited for controlling payload release from NPs [67]. Such stimuli-responsive blocks containing polymeric NPs undergo desired alterations in their characteristics, such as structural changes, switching, disassembly, or swelling upon exposure to specific environmental situations, such as specific pH, enzyme, temperature, or application of external triggers, for example, light, ultrasound, or radiations, thus releasing the cargo from the NPs to enact their anti-cancer activity [68,69,70].

### 2.1. pH-Responsive Polymeric Nanoparticles

For developing pH-sensitive polymeric NPs, two approaches have been utilized. In one of the approaches, pH-responsive, bio-degradable linkers, for example, disulfides, acetals, and hydrazones are incorporated between the core and shell of the block polymers. Upon exposure to the acidic TME, disassembly of co-polymers occurs along with their rapid degradation, thus releasing the drug inside the TME [71]. In another approach, pH-responsive, either basic or acidic segments containing the building blocks are introduced into the co-block polymer system. Upon a change in pH, protonation, ionization, and deprotonation of the polymer occurs, physical properties of the polymers are altered, such as charge conservation and chain conformation, leading to rapid release of the payload and their endosomal escape [72].

### 2.2. Thermo/Temperature-Responsive Polymeric Nanoparticles

For thermo/temperature-responsive drug delivery at the tumor site, certain polymers, such as poly(propylene glycol), poly(-isopropylacrylamide), and their derivatives have been used as they respond to the increased temperature environment that happens in tumor cells [72].

The low critical solution temperature (LCST) of such polymers is a significant trait for tumor-specific drug delivery [73]. These temperature-sensitive polymers can be complexed further with core-producing blocks via polymerization of free radicals pursued by grafting co-polymerization and hydrolysis [74]. Once they have reached the tumor site, these polymers can undergo phase transformation in an aqueous solution, this can be altered by tailoring the length of the formed block and the extent of polymerization in distinctive blocks [75]. These NPs offer stability, and a high solubility in an aqueous medium and swell in water once they fall below the LCST value due to the existence of hydrogen bonds between water molecules and the chemically functional moiety of polymers/co-polymers [76]. All this together triggers the discharge of the payload from the NPs [77].

### 2.3. Redox-Responsive Polymeric Nanoparticles

To target tumor cells containing abnormal levels of reduced glutathione (GSH), disulfide-linkers are used widely and are integrated in the co-polymer network through thiol-disulfide exchange reactions, ring-open polymerization, and controlled polymerization [78]. The polymeric NPs resulting from such reactions degrade rapidly as intracellular GSH levels rise. This is because of the reduction in integrated disulfide bonds to produce thiol groups. The process results in disassemble of the NPs, causing an instant release of the cargo into the intra-cellular compartment such as the nuclei or cytosol of the tumor cells [79].

### 2.4. Light-Responsive Polymeric Nanoparticles

Near-infrared (NIR)-sensitive polymeric NPs are promising smart cargo-carriers offering NIR-light-regulated release of therapeutic agents into tumor cells or as tumor theragnostic agents. In this methodology, a moiety sensitive to NIR light along with a chemotherapeutic is loaded inside a polymeric carrier. Upon exposure to irradiation, the NPs permit photothermal chemotherapy. Similarly, UV-sensitive and visible-light-sensitive functionalities are being explored as a technique to imbue light-sensitivity in NPs [80]. NIR-sensitive NPs have been chosen over UV–visible-responsive NPs, due to their low photo-toxicity. These NPs experience a photo-isomerization effect and a photo-chemical irreversible cleavage upon exposure to light. Due to this, the hydrophobic block of polymers is transforms into a hydrophilic block, thus disrupting the NP and promoting the release of the cargo at the tumor site [81]. The inclusion of NIR-sensitive moiety and photosensitizers also causes NPs to dissociate upon NIR-irradiation. This happens due to photo-oxidation between the NIR-sensitive moiety and photosensitizer-generated ROS that promotes instant payload discharge at the tumor site [82].

Furthermore, surface engineering of these NPs with suitable ligands allows active targeting of specific tumors, enhanced cellular uptake and cell internalization, compared to free chemotherapeutics [83]. Such approaches could prove as beneficial tools in improving therapeutic efficiency and in combating the associated systemic toxicities of chemotherapeutics. This review discusses the recent advancements in the design and development of polymeric NPs to target TNBC, and the associated challenges in their commercialization and clinical use and the possibilities of overpowering such challenges.

## 3. Polymeric Nanoparticles for Combating Triple-Negative Breast Cancer

Rational combinatorial drug therapy has an immense ability to improve the effectiveness of drugs in cancer treatment [84,85,86,87]. The concept is to utilize drug combinations with synergistic and complementary action mechanisms to inhibit different pathways involved in cancer cell survival. Modern and unremittingly developing research on cell networking routes signifies the critical role of drug release in order for ideal therapeutic outcomes [88,89,90].

MicroRNA (miRNA) is a non-coding, concise-length RNA that can efficiently control the translation of proteins. One of the exclusive characteristics of miRNA is its ability to target and direct expression regulation of multiple genes at different levels [91]. A master miRNA can work as a key knob for regulating multiple pathways.

Recently in many BC cases, miRNA-221/222 has been revealed as an important regulator involved in the initiation, epithelial–mesenchymal transition, cancer progression, and tamoxifen-induced drug resistance [92,93,94,95,96,97]. In TNBC cells, miRNA-221/222 has been found to be up-regulated, and thus it can be a potential target for monitoring/treating TNBC. In addition to this, miRNA-221/222 has also been found to be over-expressed in cancer cells resistant to PTX, signifying their participation in PTX-resistance [93].

miRNA inhibitors (miRNAi) are artificial RNA molecules containing the anti-sense sequence to a miRNA; thus, they can successfully bind to specific miRNA and inhibit its function. It has been predicted that a combination of miRNAi-221/222 and PTX could be a promising strategy for treating TNBC. However, for achieving the synergistic therapeutic benefits, both, the gene regulator and the chemotherapeutic drug should be co-transported into similar cells [86]. The designing and development of a solo transporter that can efficiently co-capture and co-transport the miRNAi and the drug to the same desired cancer site appears to be a potential method for this advanced cancer therapy [98,99]. Nevertheless, the development of such a co-transport structure that encapsulates the drug and miRNAi is a challenge in itself, mainly because of the hydrophobic nature of the drug and the hydrophilic properties of miRNAi.

NPs prepared using biodegradable and biocompatible polymers, for example, FDA-approved polyethylene glycol (PEG) and poly(lactide-co-glycolide) (PLGA) are of great interest [100]. Calcium phosphate (CaPt) is a recognized in vitro cell transfection reagent because of many reasons. In contrast to cationic polymers used for gene delivery, which possess inherent cytotoxicity and unpacking concerns as they are unable to liberate encapsulated DNA or RNA upon arrival at the tumor cells, CaPt has several benefits [101]. First of all, it is counted as a highly biocompatible reagent as it is already present inside the body [102,103], and it is easy to prepare. Another merit of using CaPt is that it is dissolved at low pH (acidic medium), causing the release of miRNAi upon pH change. When reaching acidic endosomes, miRNAi-containing CaPt dissolves, increasing the endosomal osmotic pressure, causing the rupture of its membrane and releasing miRNAi into the cytoplasm. miRNA at pH 5 releases at a much faster rate compared to pH 7 because of the faster dissolving rate of CaPt in a more acidic environment [104].

Despite their ease of synthesis, during synthesis, CaPt often precipitates and forms aggregates. This precipitation is difficult to control. However, it has been reported that by formulating CaPt in a water-in-oil (W/O) emulsion, precipitation of CaPt can be avoided [105]. A lipid coating on CaPt can further stabilize its precipitates in organic solvents [106]. The C-18 chain of lipids further offers adequate lipophilicity to the CaPt/miRNAi complex so that it can be loaded within polymeric NPs.

Zhou et al. assumed that by combining W/O emulsion and lipid, size-controllable and organic solvent soluble CaPt/miRNAi precipitates could be generated, that could be further loaded with PTX in the polymeric nanocarrier. Thus, they reported the development of a novel CaPt-polymer hybrid nano-sized carrier system for the co-delivery of miRNAi-221/222 and PTX for advanced TNBC treatment. Firstly, the miRNAi-221/222 was encapsulated in CaPt via co-precipitation in a W/O emulsion. These precipitates were then further coated using DOPA (1,2-dioleoyl-sn-glycero-3-phosphate) (an anionic lipid) to co-encapsulate lipophilic PTX outside of the hydrophilic CaPt/miRNAi-221/222 precipitate inside a solo nanocarrier. The polymeric NPs obtained by this approach had a particle size of less than 100 nm. These hybrid nanocarriers were able to co-encapsulate both the therapeutic agents within a single moiety and enhance the therapeutic efficacy of PTX.

The in vitro study was carried out at two different pH conditions. In the first 20 h of the study, around 80% of PTX was released from the NPs, irrespective of the pH of the medium. This indicates that the dissolution of PEG-b-PLGA is independent of pH. However, miRNA release happened at different and slower rates in different pH media. Only 20% release in 20 h at pH 7, whereas, at pH 5, 40% miRNA release in 20 h was observed. This is because of the enhanced dissolution rate of CaPt at acidic conditions.

Further, in in vitro studies, delivery of miRNAi-221/222 lowered miRNA-221/222 levels, thus preventing the proliferative action of miRNA-221/222. Subsequently, prevention was accomplished by the up-regulation of the miRNA-221/222 target proteins, TIMP3 (tissue inhibitor of metalloproteinase 3) and p27^Kip1^. After the successful formulation of co-transporting NPs, they were tested in vitro in the TNBC MDA-MB-231 cell line, to verify that the miRNAi-221/222–PTX-containing NPs were effective in attaining synergistic action and miRNA inhibition. The cell viability of the miRNAi-221/222–PTX NP-treated group, when checked in MDA-MB-231 cells, was markedly reduced compared to cells treated with only miRNAi-containing NPs and PTX only-containing NPs.

This indicates the synergistic action of combining a conventional chemotherapeutic drug (PTX) with RNA interference (miRNAi-221/222). This combination demonstrates the success of such a co-transportation strategy in reducing the dose of potent cytotoxic drugs without compromising their cytotoxic efficacy [107].

For accomplishing sequential release, loading different drugs into separate layers of the drug carrier is one approach, for example, using liposomes. Doxorubicin (DXN)- and Erlotinib (ETB)-loaded liposomes were prepared by loading ETB in the outer layer of the lipid and DXN inside the hydrophilic core, for achieving different release patterns. However, the issue with liposomes is their low stability which makes it problematic to fine-tune the drug release [108].

A team of researchers described the formulation and anti-cancer activity of a polymeric nanoparticle structure for the sequential delivery of these two chemotherapeutic drugs, DXN and ETB. This nanoparticle structure could co-encapsulate and co-transport this combination of drugs having distinct physiochemical properties. DXN-Hcl, due to its hydrophilic nature was conjugated with DOPA, an anionic lipid, through ionic pairing between them, forming a hydrophobic entity. ETB was encapsulated in a polyethylene glycol-b-Poly(L-lactide)-b-(PEG-b-PLA) nanoparticle. The DXN–DOPA nanoparticle was further co-encapsulated within the ETB-PEG-b-PLA nanoparticle by the process of nanoprecipitation. The conjugation of DOPA with DXN greatly helped DXN encapsulation along with a substantial reduction in the release rate of DXN. Therefore, there was a burst release of ETB and a slow, sustained release of DXN from the NPs, reported as the ideal strategy of administration for both of these drugs.

As the molar ratio of DOPA:DXN increased, the encapsulation efficiency (EE) of DXN also increased, and maximum EE was achieved when the ratio of DOPA:DXN:ETB increased beyond 5:1:3, indicating the complete complexation of DXN with DOPA. Another interesting finding showed similar a dependence of ETB encapsulation on DOPA, signifying that both DXN and ETB complex with DOPA. Although the EE of DOX was almost double the EE of ETB. However, the EE of ETB with DOPA was about 90%, in the absence of DXN, signifying the interference of DXN in the complex formation of ETB and DOPA. Additionally, the molar ratio of 1:1 of the drug to DOPA was enough to give an EE of ~90% for both drugs (either DXN or ETB).

In vitro efficacy of this sequential nanocarrier was substantiated and its potential applicability was validated in vivo through the fluorescence imaging of tumors showing a high accumulation of polymeric NPs. An in vitro cytotoxicity assay was conducted on the basal-like 1 TNBC cell model, MDA-MB-468 cell line. To assess the reported enhancement in the efficacy of the consecutive and controlled release of DXN and ETB, the MDA-MB-468 cell line was treated with NPs of varying molar ratios of DOPA:DXN:ETB. An MTS assay was performed to evaluate cell viability with one-day and two-day incubation periods after adding DXN. The cytotoxic effect of the prepared polymeric NPs was markedly improved with increased molar ratios of DOPA:DXN:ETB for two days. This improvement in the cytotoxic effect is due to the tumor cells’ sensitization by the chronological release of ETB, followed by the release of DXN. Among all the molar ratios evaluated, the ratio of 5:1:3 of DOPA:DXN:ETB was considered the ideal ratio providing maximum EE. With a molar ratio of 2:1:3, the EE of ETB was not optimal to inhibit EGFR. Increasing the ratio of DOPA beyond five (10:1:3) showed no increase in the loading of ETB and DOX, exhibiting the same cytotoxic efficacy as that of the ratio 5:1:3.

In vitro toxicity of the lipid (DOPA) and both the polymers PEG–PLA was also checked. Indeed, none of them showed any type of toxicity to the cell line.

A Fluorescent bio-distribution test was conducted in an orthotopic breast tumor model. Mice were administered with NPs and were then imaged at the 1st, 4th, and 24th hour. NPs were seen to be accumulating inside the tumor cells immediately within 1 h of administration and accumulation was continued even at the 24th hour. Even though the NIFR intensity was considerably high in the 1st hour, it decreased with time, signifying the steady excretion of NPs accumulated in the liver. At the 24th hour, the tumor showed the highest NIFR intensity, throughout the whole body.

After 15 days of injection, the animals were sacrificed, and major organs were then harvested for ex vivo imaging. The tumor still depicted the highest fluorescence intensity among all organs, representing the accumulation of NPs even after 15 days of treatment and the clearance of NPs from major organs, including the kidney, spleen and liver [109].

Targeting receptors over-expressed on the cell surface via ligand-modified NPs has become a popular approach for delivering and internalizing drug-containing NPs into TNBC cells via several endocytosis pathways. Small-sized NPs, preferably of 20–30 nm in size, are desired to target receptors and be internalized into cells, particularly by clathrin-dependent endocytosis [110]. Other NPs have been designed to internalize through the caveolae-dependent pathway, as this signaling pathway dodges fusion and lysosome formation, thus evading NP degradation [111]. However, Bobrin et al. in their research work have shown that even without cell binding receptor compounds, an asymmetric polymeric tadpole-shaped nanostructure coated with a thermo-sensitive polymer, poly(N-isopropyl acrylamide) resulted in tumor selectivity as well as a higher uptake into the MDA-MB-231 TNBC cell line. Observations were made that, NP surface coated with poly(N-isopropylacrylamide) along with the unique tadpole shape exhibited about 15 times greater NP cellular uptake in comparison to spherical-shaped NPs composed of the same polymer. The mode of entry was most preferably by phagocytosis. Further, tadpole-shaped polymeric nano-micelles were loaded with DXN and exhibited a ten-times reduction in the IC_50_ of DXN, in contrast to free DXN. Further observations showed that cancer cell death occurred through late apoptosis, this further safeguard healthy cells from the innate immune system of the body. Overall, the results demonstrated that by modifying the polymeric conformation, chemical composition and by providing an asymmetric shape to the NPs, the selectivity as well as the efficacy of the chemotherapeutics could be improved and such alterations could allow designing NPs for required cancer outcomes [112].

TRAIL or tumor necrosis factor-related apoptosis-inducing ligand plasmid (p-TRAIL) is a type 2 transmembrane protein of the TNF family known for inducing apoptosis in several cancers without affecting normal cells [113]. The extrinsic pathway is promoted by TRAIL-receptor complex formation leading to caspase-3 cleavage and subsequent activation of caspase-8. Soluble recombinant TRAIL revealed excellent pre-clinical anti-cancer activity. However, clinical responses were rare, possibly because of poor involvement of the extrinsic pathways [114].

To resolve this issue, embelin (EBN), a natural benzoquinone derivative obtained from the fruits of *Embelia ribes* has been used. It shows pharmacological and therapeutic activity against inflammation, fever, diabetes, cancer, etc., EBN promotes the extrinsic pathway by facilitating caspase-3 activation [115]. It is a well-recognized inhibitor or XIAPs (X-linked inhibitor of apoptosis proteins); therefore, its anti-cancer activity is because of its inhibitory action against binding between caspases and XIAPs [116].

Co-delivery of EBN and TRAIL could be a potential approach for synergistically inhibiting XIAPs, activating caspases and inducing apoptosis. However, to encapsulate this synergistic combination of a gene and a chemotherapeutic, developing a single vehicle for co-transporting a nucleic acid and a drug is needed. To resolve this, Xu et al. used two widely known cationic polymers, PEI and PBAE (poly-beta-amino ester) to construct TRAIL- and EBN-encapsulating polymeric NPs [117,118]. PBAE is known for its pH sensitivity, good transfection ability and low toxicity. It is lipophilic in neutral pH but converts to a hydrophilic nature in acidic pH. This facilitates the release of PBAE-encapsulated drugs into the tumor microenvironment [119]. Furthermore, to limit the systemic toxicity and enhance the targeting ability of PEI, a hyaluronic acid (HA) coating on PEI–PBAE NPs was conducted [120,121].

Various studies report the application of a naturally occurring polysaccharide, hyaluronic acid (HA). It is a biocompatible, non-immunogenic, non-toxic, non-sulfated glycosaminoglycan, made up of repetitive units of N-acetyl-D-glucosamine and D-glucuronic acid. HA is a specific ligand to the membrane glycoprotein CD44, a cell surface receptor overexpressed in TNBC and participant in different malignant cell activities [122].

**Figure 1:**
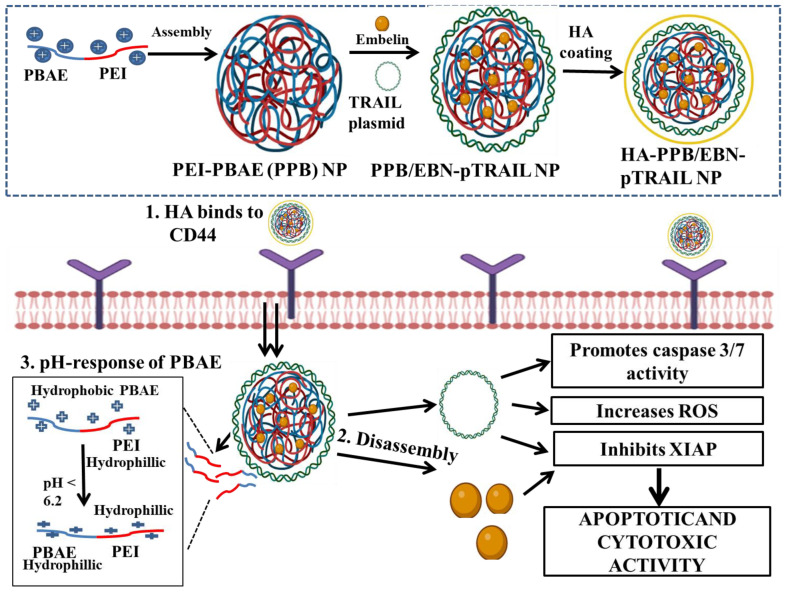
Schematic representation of the anti-cancer activity of embelin and TRAIL-encapsulated, hyaluronic acid-coated, PBAE–PEI nanoparticles (HA-PPB/EBN-pTRAIL NP). The co-delivery of TRAIL and embelin prompted caspase 3/7 activity, elevated the level of ROS and suppression of apoptosis-related protein.

Due on this, HA-coated PEI–PBAE NPs encapsulating a TRAIL plasmid and EBN were developed (HA-PPB/EBN-pTRAIL NP) (Figure 1). Coating with HA successfully avoided aggregation between cationic polymeric NPs and serum components as demonstrated by the improved stability and reduced hemolysis due to NPs. Moreover, HA-PPB/EBN-pTRAIL NPs depicted pH sensitivity, favorable condensation activity of pTRAIL, and significant entrapment efficiency of EBN. Additionally, when checked in CD44 overexpressing MDA-MB-231 cells in vitro, HA-PPB/EBN-pTRAIL NPs resulted in elevated cellular uptake because of the interaction between CD44 and HA. Co-delivery of pTRAIL and EBN remarkably improved the apoptotic and cytotoxic activity by promoting caspase 3/8 activity, increasing ROS and inhibiting XIAPs. Overall, HA-PPB NPs present themselves as a potential carrier for co-transporting therapeutic genes along with chemotherapeutics. Additionally, this synergistic combination of pTRAIL and EBN can be used as a potent therapy for TNBC treatment [117].

Similar to TQN, piperine (PPN) is another phytochemical possessing anti-cancer activities. PPN is a pungent alkaloid and active component of *Piper nigrum* (black pepper) with anti-cancer and other pharmacological properties [123]. Several studies also testify to the cytotoxic and anti-proliferative effects of PPN on different types of cancer, including mammary cancer [124,125,126,127]. However, its clinical application is also hindered by its hydrophobicity [128]. PLGA and PEG have been frequently used to design co-polymeric NPs as they possess admirable biocompatibility and hydrophilicity and PEG also protects the NPs from instant opsonization and elimination through phagocytic cells [129]. Thus, a group of scientists decided to produce PPN-loaded–PLGA-mPEG co-polymer NPs (PPP-NPs) via thin-film hydration and the single-emulsion solvent extraction method. NPs produced using the single-emulsion solvent extraction technique yielded NPs of large size and irregular shape. On the other hand, NPs produced via the thin-film hydration technique produced NPs of desired size (32–82 nm) and regular shape [130].

The desired size of NPs helps in easy passive diffusion of NPs into the TME while dodging retention in the spleen, liver, and kidney, thus avoiding toxic side effects [131]. Therefore, NPs yielded via the thin-film hydration technique were selected for further research. The efficacy of PPP-NPs in inhibiting TNBC growth was checked in human MDA-MB-468 cells and breast ductal carcinoma cells BT-549 and compared to the efficacy of free PPN via MTT assay. Results showed that free PPN and PPP-NPs exhibited dose-dependent, equivalent inhibitory effects on the tumor cell growth of both the cell lines, signifying that the PPN encapsulation into polymeric NPs did not lower the anti-cancer efficacy of PPN [130].

Poly(N-(2-hydroxypropyl)-methacrylamide) or pHPMA is a polymeric block extensively used in drug delivery and various other biomedical therapies. It is known for its numerous advantageous properties such as hydrophilicity, biodegradable and biocompatible nature, and its ability to conjugate with major ligands for tumor cell targeting [132,133]. As already discussed, surface modification with PEG promotes NPs ability to circulate longer in blood, resulting in their higher accumulation inside tumors [134].

The PEGylated NPs can successfully escape opsonization and thus macrophage recognition resulting in a longer stay of NPs in the blood.

Taking advantage of the properties of both the polymers, Bobde and team synthesized HPMA-b-methoxy PEG (mPEG) polymers using a different initiator to monomer feed ratios. Although the hydroxyl moiety of pHPMA makes it hydrophilic, the polymethacrylate group of HPMA makes the HPMA-b-mPEG block hydrophobic. The polymers with altered HPMA chains self-assembled into micelles to load the chemotherapeutic moiety, DXN. The developed polymer was characterized using NMR (nuclear magnetic resonance), IR (infrared spectroscopy), GPC (gel permeation chromatography), CMC (critical micelle concentration), etc. The results showed that the micelles formulated using a polymer with HPMA:mPEG and drug:polymer ratio of 175:1 and 1:10, respectively, resulted in NPs with the particle size distribution of a narrow range and high drug loading compared to other synthesized ranges of polymers. Micelles containing the drug were characterized thoroughly and their stability was analyzed for 3 months. At the end of 3rd month, DXN retention in selected micelle was 94.39%. Additionally, the particle size and zeta potential of the selected formulation were unaffected when stored at 4 °C. Furthermore, the micelles showed stability for up to 72 h in DMEM containing serum. An insignificant rise in PDI (polydispersity index) of micelles was noticed but no remarkable change in size occurred after incubating the micelles in 10% FBS-containing DMEM medium for 72 h at 37 °C.

The DXN-loaded micelles released DXN slowly and effectively in a pH-responsive manner, beneficial for tumor-targeted drug delivery. The polymeric micelles efficiently penetrated into the 4T1 murine, MCF-7 and MDA-MB231 human breast cancer cell line in a time-dependent manner, inducing higher cell cytotoxicity compared to free DXN, in vitro. Pharmacokinetic estimation and hemocompatibility assays were estimated in Wistar rats.

The pharmacokinetic assay showed that NPs efficiently transported drugs into systemic circulation and prolonged its blood circulation. In conclusion, the study represents the work on developing easy-to-formulate, economical, easily scalable and stable DXN-loaded polymeric micelles that is a promising therapeutic option for treating solid tumors, especially TNBC [135].

Dopamine is a melanin-mimicking muscle-adhesive protein that can self-polymerize into surface-adherent poly-dopamine (PDA) films [136,137,138,139]. Recently, PDA has gained popularity as a potent photothermal therapeutic moiety because of its distinctive characteristics such as great biodegradability and biocompatibility, high photothermal conversion ability and strong NIR light absorption [140,141,142].

**Figure 2:**
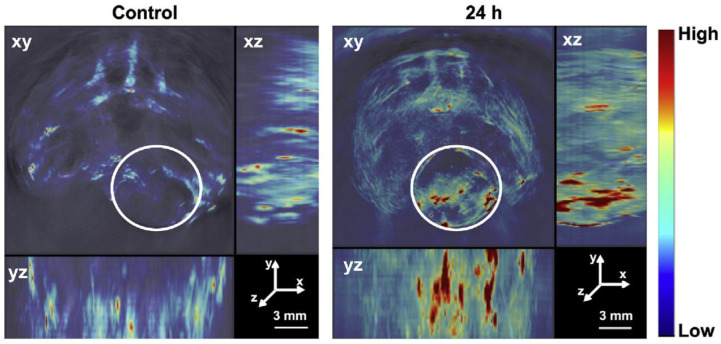
Photo-acoustic images showing tumor accumulation and photothermal efficiency of DPS-NP/PDA after 24 h of nanoparticles administration in tumor-bearing mice. Reproduced with permission from reference [143].

A group of scientists were interested in the coating of PDA in thermo-sensitive polymeric NPs make them capable for photothermal therapy under NIR radiation and whether it also protects the drug encapsulated in the NP from initial burst release. So they formulated polymeric core–shell structure NPs using a novel amphiphilic co-polymer poly(2-(2-methoxyethoxy) ethyl methacrylate co-oligo (ethylene-glycol) methacarylate-co-2-(dimethylamino) ethyl methacrylate-b-poly(lactide-co-glycolide) [143]. This co-polymer combined the merits of PEG with poly(N-isopropyl acrylamide), presenting a thermo-sensitive nature, low immunogenicity and thus low toxicity [141]. By slight modifications during polymer synthesis, the LCST of the selected co-polymer can be altered to be slightly elevated above normal human body temperature; therefore, even a slight increase in temperature through external heating can lead to NPs collapse for efficient release of the drug. Initially, chemotherapeutic agents PTX and DXN were encapsulated into a lipophilic layer and a lipophobic core, respectively. Survivin siRNA was adsorbed onto the surface of the NPs via electrostatic bonding (DPS-NPs). The NP-encapsulated drugs were then coated with an outer layer of PDA (DPS-NP/PDA). The spatio-temporal release of both the drugs and the remarkably enhanced therapeutic effect due to combinatorial drug delivery through DPS-NP/PDA against TNBC was investigated. Evaluating the deposition ability of DPS-NP/PDA inside tumor tissues, NPs were tracked in the tumor cells through multispectral opto-acoustic tomography (MSOT) because of its high NIR absorption and photothermal conversion capacity. After 24 h of DPS-NP/PDA injection into mice, diverse photo-acoustic signals were seen in the tumor region. Contrary to this, limited signals were detected in the tumor region of the mice, before treating them with DPS-NP/PDA (Figure 2).

Surface-modified PDA prevented burst release of drug from the nanocomposite. The formulated NPs produce sufficient heat required for photothermal therapy, under localized NIR irradiation and thus precise thermo-dependent drug release was achieved for gene therapy and chemotherapy ultimately causing TNBC cell regression and improved chemosensitivity [143].

A group of researchers also developed micellar-like NPs (MR-NPs) loaded with the anti-cancer drug DCL using redox-sensitive cross-links fabricated into a terpolymer to deal with the abnormal biology of TNBC. The MR-NPs were designed using terpolymer poly(ethyleneglycol)-b-poly(lactide)-co-poly(N3-alpha-ε-caprolactone) with a di-sulfide linker trailing from the caprolactone to cross-link adjacent chains. This terpolymer contained both poly-caprolactone and poly-lactide to maintain a balance of reducing agents essential for ensuring stability along with the rapid breakdown of micelles and thus drug release from them as soon as they enter breast cancer cells including elevated levels of reductive agents. Empty MR-NPs did not exhibit any cytotoxicity in 2D monolayers of the TNBC cell line (MDA-MB-231), breast cancer cell line (MCF7) and normal breast cell line (MCF10A) in vitro. However, DCL-loaded MR-NPs showed higher cytotoxicity against both the breast cancer and TNBC cell lines that contained elevated levels of intra-cellular GSH. Non-cross-linked and cross-linked MR-NPs depicted high cellular uptake in monolayers and tumor spheroids of TNBC cells and cancer-linked fibroblasts in a concentration-dependent manner. DCL-loaded-cross-linked MR-NPs exhibited the greatest efficacy towards 3D spheroids of TNBC, they also showed an enhanced level of apoptotic activity as measured through Annexin V/PI evaluation and elevated activity of caspase 3 and 7 in the MDA-MB-231 cell line compared to DCL-loaded non-cross-linked MR-NP-treated cells (control group). Altogether, the results established that the MR-NPs formulated using terpolymer exhibited greater efficacy in vitro, in both 2D and 3D TNBC models and thus can be an effective and potential drug-delivery system for further clinical application in TNBC treatment [144].

The tumor cell-penetrating peptide, iRGD (CRGDKGPDC) comprises of an RGD motif which interacts specifically with overexpressed integrin αvβ3 in cancer endothelial cells [145]. Successive cleavage of iRGD reveals the CendR (RGDK) motif which has a greater affinity towards binding with neuropilin-1 (NRP-1) receptors. This receptor–ligand binding affinity encourages endocytosis and transcytosis, promoting tumor penetration [146]. Therefore, surface modification of drug-loaded polymeric NPs with the iRGD peptide enhances their penetration into solid tumors [147].

To utilize this scheme, Mamnoon et al., designed self-assembled hypoxia-sensitive polymersomes (PMs) made up of hypoxia-sensitive PLA-diazobenzene-PEG di-block co-polymers. Further, iRGD was conjugated onto the surface of PMs assembly to prepare iRGD-conjugated hypoxia-sensitive polymersomes (iRGD-PMs). DXN was loaded for chemotherapeutic activity and both non-targeted and targeted DXN-containing hypoxia-sensitive PMs (DXN-iRGD-PMs and DXN-PMs) were prepared and their efficacy was checked in vitro as well as in vivo. Results confirmed the hypoxia-sensitive nature of PMs as less than 30% of DXN was released from the PMs in 12 h under a normoxic environment, i.e., 21% oxygen, whereas DXN release increased significantly up to 95% under hypoxic conditions, i.e., 2% oxygen. The targeted PMs remarkably reduced cell viability in the monolayer and spheroid cultures of TNBC under hypoxic conditions contrary to normoxia. Further, in vivo studies performed in MDA-MB-231 cell lines containing female nude mice displayed a significant reduction in tumor growth. Altogether, DXN-iRGD-PMs depicted potential anti-cancer activity in monolayer, spheroid, and xenograft models of TNBC [148].

Combination chemotherapy is clinically used to lower the risk of post-operative reoccurrence and metastasis or to advance the possibility and rate of success of surgery, pre-operatively. Biguanides, such as phenformin and metformin, along with anti-diabetic properties, help in decreasing the risk of tumor and cancer occurrence [149,150]. In recent years, various guanidine-derived compounds have been synthesized that display anti-cancer activity [151,152]. The presence of the cationic group in the guanidine aids in the quick penetration of the carrier–drug complex into the tumor cells [153]. One such example is phospholipids and cholesteryl biguanide conjugates surrounding siRNA NPs [154].

Magnolol (MGL) is another polyphenol obtained from the bark of the plant *Magnolia officinalis.* It has many pharmacological activities such as anti-cancer, anti-oxidant, anti-inflammatory, neuroprotective activity, etc. [155]. It has low toxicity and a good safety profile [156]. MGL hinders tumor cells growth in different cancers, such as A549 non-small lung cancer cells [157], HCT116 colorectal cancer cells [158], T24-human bladder cancer cells [159], and GBC-human gall bladder cancer cells [160].

MGL is a potential inhibitor of tumor cell proliferation, angiogenesis, differentiation and migration and a potent inducer of apoptosis and for reversing MDR [161]. However, it is a hydrophobic compound.

Cholesteryl biguanide-conjugated hydrochloride (CBCH) can self-assemble itself into a NP and can efficiently encapsulate MGL-like compounds. Thus, the combination of MGL and CBCH may bring a synergistic inhibitory action on cancer cell growth. Based on this, Wang and team developed a NP formulation using CBCH as a primary carrier of MGL (MGL–CBCH NPs) into the tumor cells and then further coated the CBCH NPs with PLGA-mPEG to extend the blood circulation time of the NPs [162].

As previously reported, sigma receptors have been found to be overexpressed on the 4T1 cell surface [163], so the team conjugated its ligand aminoethyl anisamide-polyethylene glycol (PEG)-poly(lactide-co-glycolide) or AEAD-PEG-PLGA on to the external surface of the formulated NPs to increase the tumor-targeting ability of the NPs. They optimized the CBCH synthesis, assessed the ratios of selected compositions of the NPs in vivo and examined the effect of the selected combination on 4T1 cell growth, both in vitro and in vivo.

NPs composed of a 1:4 weight ratio of mPEG-PLGA to AEAD-PEG-PLGA, showed the highest accumulation inside the tumor cells and a combination of MGL and CBCH displayed a noticeable synergistic inhibitory activity against 4T1 cells. Results of the in vitro assay reported that the selected NPs exhibited the highest cell uptake, greatest apoptosis rate and remarkable inhibition of monoclonal formation and cell migration. When administered intravenously into the 4T1-containing xenograft mice model, NPs inhibited the growth of the tumor and showed no obvious side effects. Results of Western blotting confirmed that the NPs regulated the levels of p-AMPK, p-AKT, and p53, present in the tumor tissues. Furthermore, cell apoptosis occurred in the same region of TUNEL-stained tumors and H&E-stained tumors, exposed to the NPs. Overall, this NP system offers a potent combination of the drug for treating TNBC [162].

In another study, Basu et al. developed NPs of metformin using graphene oxide (GO) [164]. GO is the oxidation product of graphene and is gaining tremendous response for cancer nano-therapies. Because of its easy functionalization ability and high surface area to volume ratio, it is extensively being used as a drug carrier [165,166]. The functional groups present on the GO surface permit its conjugation with different polymers [167]. Besides being an outstanding drug carrier, recent reports suggest an anti-migratory activity of GO [168] along with selective targeting and proliferative arresting of CSCs [169]. Therefore, researchers developed metformin-encapsulating GO NPs and further anchored it to polymeric NPs designed using PLGA-PEG block co-polymers. The whole assembly was further coated by HA (Met-GO-PP-HA). This novel system fulfilled the responsibility of actively targeting TNBC cells and solved the issue of the aqueous solubility of metformin. The NPs showed remarkable cell death and inhibited migration of TNBC cells efficiently, compared to the non-targeted formulation (Met-GO-PP-NH_2_). Further, the molecular mechanism was delineated, both in vitro and ex ovo. 4T1 murine mammary tumors were developed in Balb/c mice and the activity of Met-GO-PP-HA NPs was validated in vivo. The NPs targeted miR-10b by restricting the translocation of the nuclear transcription factor, NFkB-p65 inside the cell nucleus, thus potentiating the anti-tumor effect. miR-10b down-regulation, further up-regulated the tumor suppressor gene, PTEN, which then influenced the downstream regulator, pAKT-473, causing cellular apoptosis in the TNBC cells. In addition to this, miR-10b led to PTEN up-regulation and down-regulation of integrin beta-1 and pFAK, contributing to the inhibition of cell migration. These NPs also inhibited mammosphere formation, reduced stemness markers such as sox2, nanog, oct4, and increased the expression of E-cadherin more efficiently compared to the control group. Collectively, the targeting of NFkB-p65 and miR-10b by Met-GO-PP-HA NPs resulted in the effective treatment of TNBC and CSCs [164]. Table 1 summarizes different polymeric nano-formulations for combating TNBC and its associated challenges.

## 4. Polymeric Nanoparticles for Triple-Negative Breast Cancer Immunotherapy

Over the past years, cancer immunotherapy (IT) has gained much attention for advanced TNBC treatment [179,180,181]. Sustained progression-free survival has been achieved in patients suffering from advanced breast tumors through pembrolizumab + chemotherapy [182]. However, regardless of promising results, poor immunogenicity and immune-suppressive TME are unavoidable hurdles of almost all immunotherapies while treating TNBC [183].

Charge-reversal NPs are widely used because of their various advantages including escape from reticuloendothelial system clearance, improved tumor-specific NPs accumulation, and enhanced cell uptake and penetration [184,185]. An example of this is a charge-reversal and self-amplifiable prodrug polymeric micelle, constructed to treat multi-drug resistance (MDR). The zeta-potential of the formulated micelles switches from −ve to +ve resulting in a better cellular entry in an acidic TME [186].

Nucleic acid (NA) drugs used in cancer IT, such as CpG oligodeoxynucleotides [187], polyinosinic–polycytidylic acid (poly(I–C)) [188], etc., exhibit excellent tumor immunogenicity [189,190]. Cationic polymers are being used to condense such drugs into NPs through electrostatic bonding as this polymer/NA drug complex facilitates steady loading and successful internalization into cancer cells without dissociating into the cytoplasm [191,192].

Similarly, Fang and team developed a NIR light-regulated charge-reversal NPs (NCRNPs) to co-transport photosensitizer chlorin e6 (C-e6) and poly(I–C), aTLR3 agonist for enhanced photodynamic IT against TNBC. The NPs underwent self-assembly because of the presence of Fmoc (9-fluorenyl methoxy carbonyl)-KCRGDK-phenylboronic acid (Fmoc-K-PBA), PVA (polyvinyl alcohol) and cationic PEI-derived C-e6 (PEI-C-e6) that facilitated successful loading of poly(I–C) with an anionic nature [170].

Upon NIR irradiation, NCRNPs stimulated ROS production and consecutively induced phenylboronic ester group cleavage present between PVA and Fmoc-K-PBA [193]. Additionally, after NIR irradiation, the presence of anionic oxygen and anion boron led to +ve to −ve charge reversal of the NCRNPs, in turn releasing poly(I–C). Furthermore, NCRNP disassembly promoted reduction in the TME as the disulfide bond between Fmoc-K-PBA broke.

Results of in vitro and in vivo studies showed that NCRNPs are distributed inside the tumor cells, activate DCs, induce ICD in cancer cells, and encourage anti-tumor immunity along with the ultimate inhibition of TNBC cell progression. An in vivo study was conducted using a 4T1 TNBC cell-carrying female Balb/c mouse model. The treatment was started after the tumor volume reached up to 100 mm^3^. Animals were subjected to different formulations and NIR lights, in selected groups, twice a day. Although NCRNPs + PDT completely disrupted the tumor in the initial days of treatment, it relapsed rapidly after 111 days, as observed by the elevated tumor growth kinetics. In the NCRNP–poly(I–C) + NIR irradiation-treated group, a significant anti-cancer effect was observed with no relapse. The treatment with NCRNP–poly(I–C) + NIR delayed 97% of cancer cell growth compared to the control group, indicating an enhanced anti-tumor efficacy [170].

The research agrees that stimulation of anti-cancer immunity at the cancer site may lead to systemic side effects [194,195]; thus, the anti-cancer activity of NCRNP–poly(I–C) was examined using an abscopal tumor model. The primary tumors were treated two times a day and distant tumors were then inoculated. It was observed that abscopal tumors were formed earlier in animals treated with NCRNPs or PBS compared to the NCRNP–poly(I–C) + NIR group. Eighty percent of animals were found to be free of abscopal tumor formation after NCRNP–poly(I–C) + NIR therapy. The charge-reversal NPs thus provides an innovative strategy for the controlled release of nucleic acid-based immune modulators that may improve photodynamic cancer IT of TNBC [170].

Studies have revealed that TNBC has many tumor-infiltrating lymphocytes (TILs) and also overexpress programmed cell death-ligand 1 (PD-L1), indicating that immunotherapy could be a promising approach for treatment [196,197]. Blocking PD-L1/PD-1 in TNBC therapy can have a lasting response; however, most PD-1/PD-L1 blocking therapies used have a low rate of response in TNBC [198,199]. CD155 has also been seen to be highly expressed in various tumors, whereas it is absent or has low expression in normal tissues [200]. CD155 intrinsically promotes metastasis of tumors, while extrinsically, it regulates the immune activity by being a ligand of co-inhibitory receptors, TIGIT and CD96, and co-stimulatory receptor, DNAM-1 present on CD8+ T cells [201]. DNAM-1, a co-stimulatory receptor, is also found to be highly expressed in CD8+ cells. The interaction of DNAM-1 with CD155 can further accelerate the activation of CD8+ cells which in turn accelerates tumor cell killing [202,203]. Results from both, Clinical samples and pertinent tumor mouse models verified that loss of DNAM-1 restrains functions of CD8+ T cells and restricts its efficacy in cancer therapy [204]. On the contrary, TIGIT and CD96, co-inhibitory receptors, are found to be up-regulated in the later stages in the CD8+ T cells, and the interaction of TIGIT and CD96 with CD155 has been reported to prevent the activation and cytotoxic activity of T cells [205]. Furthermore, CD96/TIGIT has a greater affinity for CD155 compared to DNAM-1 and thus CD96/TIGIT competes with DNAM-1 for binding to CD155 [206].

**Figure 3:**
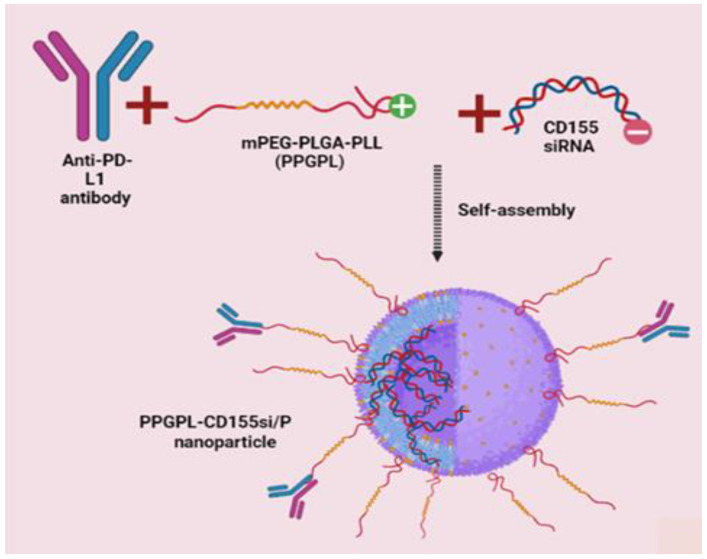
Schematic representation of designing mPEG-PLGA-PLL (PPGPL) polymeric nanoparticles loaded with CD155siRNAand covered with PD-L1 antibodies on the exterior surface to block CD155 and PD-L1 in a spatio-temporal manner.

A group of scientists in their study confirmed that both, PD-L1 and CD155 are overexpressed on TNBC cells. Additionally, when they examined the receptors of CD155 and PD-L1 over time in the TILs of TNBC, they witnessed that initially DNAM-1 and PD-1 receptors were up-regulated, whereas TIGIT and CD96 were up-regulated in exhausted CD8+ TILs. Considering all these observations, these scientists decided to develop a mPEG-PLGA-PLL (PPGPL) polymeric NP encapsulating CD155 small-interfering RNA (siRNA) (CD155si), covered with PD-L1 antibodies on the exterior surface (PPGPL-_CD155si_/P) to block CD155 and PD-L1 asynchronously in a spatio-temporal manner (Figure 3) [171]. Anti-tumor immunotherapy research currently is focused on a static time point or solo pathway of the tumor microenvironment (TME), contrary to this, these scientists investigated the effect of cross-talk and dynamic expression of the TIGIT/CD96, PD-L1/PD-1, CD155/CD96, CD155/DNAM-1 axes on the anti-tumor function of CD8+ T cells in the TNBC TME.

A positive charge on PLL can improve the loading of PLL by successfully binding PLL to the RNA having a negative charge, thus acting as a penetration enhancer, with increased transfection efficiency. Therefore, PEAL having a positive charge acts as the spine of the whole assembly, it has been validated to have an excellent loading capacity for siRNA, high passive tumor-aiming targeting capability and great biocompatibility [207]. PPGPL-_CD155si_/P promoted CD8+ T cell immune supervision against 4T1 tumors in the early stages, whereas in the late stages, it inhibited CD8+ T cells to inhibit the immune escape of 4T1 tumors. In addition to this, the combination of tumor-targeted CD8+ T cells and PPGPL-_CD155si_/P promoted 4T1 cell immunogenic cell death (ICD) to encourage this immune checkpoint regimen further. To verify the specific binding of the NPs, 4T1 cells were first subjected to incubation with PBS, PPGPL-_cy5-CD155si_/P, PPGPL-_cy5-CD155si_/I at 4 °C for 30 min and then analysis by flow cytometry of these cells was conducted. Cells bound to the NPs were Cy5 positive. Results exhibited the binding of only PPGPL-_cy5-CD155si_/P with the 4T1 cells, indicating that NPs can bind to α-PD-L1 effectively without eluding its binding specificity. The release profile of siRNA showed that within the initial 24 h, it was released rapidly (38.34%) from the PPGPL-_cy5-CD155si_/P, then later it showed a slower release with up to 85% in 120 h, exhibiting that the PPGPL-_cy5-CD155si_/P NPs have a controlled release pattern [171].

The development of resistance and poor prognosis of TNBC has been attributed to its strong metastatic nature. As reported, CD155 has a critical role in tumor metastasis and its activity has been examined by wound healing and transwell migration assays. It was investigated whether the PPGPL-_CD155si_/P NPs mediated CD155 knockdown could inhibit cell invasion and migration in 4T1 cells. The assay indicated that metalloproteinase-2 (MMP-2) and metalloproteinase-9 (MMP-9), tumor metastasis marker proteins were down-regulated significantly after the treatment with PPGPL-_CD155si_/P NPs, verifying that the PPGPL-_CD155si_/P NPs were successful in inhibiting cell invasion and migration efficiently [171].

Furthermore, when investigated in a 4T1 TNBC orthotopic tumor model, PPGPL-_CD155si_/P exhibited excellent targeting of TNBC cells, additionally inducing a CD8+ TIL-dominant anti-tumor immune response to effectively hamper TNBC metastasis and progression with the least toxicity. In bio-distribution studies, the distribution of NPs was traced using cy7-siR. The fluorescence signals of cy7-siR were monitored and revealed that the fluorescence signal was predominately traced to the abdomen; however, they weakened gradually and eventually disappeared after 24 h. Inverse to this, high fluorescence intensity was observed at the tumor site, which did not weaken even after 48 h. In addition to this, the pharmacokinetic results showed that PPGPL-_CD155si_/P protected against RNA degradation leading to its long-term circulation. The half-life of PPGPL-_CD155si_/P and free siRNA were found to be 13.73 ± 0.93 h and 1.26 ± 0.45 h, respectively. The therapeutic efficacies of PPGPL-_CD155si_/P were also evaluated in the orthotopic tumor model by analyzing the tumor growth and spontaneous metastasis to the lungs.

Unbound siCD155 was unable to retard tumor growth as it was rapidly cleared by RNA in vivo, whereas PPGPL NPs encapsulating siCD155 showed a therapeutic effect. Additionally, TUNEL staining of the tumor tissues established that the rate of apoptosis in the PPGPL-_CD155si_/P-treated group was significantly greater compared to the other groups.

Overall, the study unveils a potential combination strategy for immunotherapy treating PD-L1/CD155+ TNBC. Additionally, this is a promising candidate for wide application in CD155 and PD-L1 co-expressing cancers [171].

## 5. Polymeric Nanoparticles Combating Cancer-Stem Cells in Triple-Negative Breast Cancer

Several tumors comprise functionally and phenotypically heterogeneous masses of tumor cells that differ in angiogenic and metastatic potential and drug-resistance. Some types of cancers develop cancer-stem cells (CSC), causing the heterogeneously diverse cancer cells as a result [208]. Increasing reports indicate the presence of distinctive phenotypes of CSC, high tumorigenicity and self-renewing abilities, which is the driving force of tumor origin, progression, metastasis and drug-resistance development in these kinds of tumors [209]. Additionally, CSCs are themselves commonly found to be drug-resistant, thus they dodge traditional chemotherapies and reproduce tumors causing the reoccurrence of cancer, which is the main cause of high mortality in cancer patients. Therefore, effective and efficacious chemotherapy for these types of tumors should be able to eradicate these CSCs effectively, thus preventing the relapse of cancer [210].

To overcome this issue, Zhao and colleagues proposed an approach of employing polymeric micelles made from Pluronic block co-polymers, encapsulating DXN. These amphiphilic micelles have a distinctive characteristic of chemo-sensitizing multi-drug resistant cancers (MDRC) by various mechanisms: (i) by inhibiting mitochondrial respiration, ATP synthesis, and hindering ABC transporter-drive drug efflux, (ii) by improving pro-apoptosis in MDRC cells. Optimal Pluronic with specific compositions can even prevent drug resistance development and also inhibit cell metastasis [172].

A polymeric micelle preparation of DXN formulated using a mixture of Pluronic F127 and L61, SKC1049 (formerly known as SP1049C), was assessed in phase I and II clinical trials in patients suffering from inoperable-metastatic esophageal and gastro-esophageal junction adenocarcinoma. This formulation produced a great objective rate of response (43%) and an improved median survival rate (10 months) [211]. Zhao and colleagues, in one of their studies described the tumorigenic action of SKC1049 against highly aggressive murine leukemia cells via CSC targeting. Further, they extended their work by evaluating SKC1049 ability in eradicating CSCs from TNBC cells. SKC1049 involved the use of the solo chemotherapeutic drug, DXN along with GRAS (generally recognized as safe) excipients.

Basal MDA-MB-468 and claudin-low MDA-MB-231 TNBC cell lines were used to demonstrate that SKC1049 was effective in reducing the CSC population and thus reducing tumorigenesis both in vitro and in vivo. In both the cell lines, ESA+CD44^high^ CD24^low^ cells were isolated that showed resistance to DXN and exhibited CSC properties, such as high invasion, migration, colony formation in vitro and extreme tumorigenicity in vivo in comparison to their parent or non-CSC (ESA-CD44^low^ CD24^high^) counterparts. Contrasting to the MDA-MB-468 basal type TNBC cell line, the MDA-MB-231 cell line is a claudin-low cell line and it usually has a poor reaction to chemotherapy. Such types of tumors are distinctive because of the low-expression of claudin-3 and claudin-4, down-regulation of the Ki67 proliferation marker, up-regulation of markers linked to epithelial–mesenchymal transition and enrichment of characteristics correlated with mammary CSCs which involve the CD44^+^ CD24^−/low^ phenotype. MDA-MB-468 does not exhibit such features, therefore both the cell lines differ in phenotypic and molecular behavior. Additionally, MDA-MB-231 is an excessively metastatic cell line, although MDA-MB-468 is not.

Another distinctive feature between both the cell lines witnessed during this work was their difference in depletion of ATP induced by the SKC polymer. The parental cells of MDA-MB-468 as well as the isolated CSCs (ESA+CD44^high^ CD24^low^) and non-CSCs (ESA-CD44^low^ CD24^high^) population of the MDA-MB-468 cell line showed intense ATP reduction in the presence of the SKC polymer with 0.02% to 0.2% weight. Contrary to this, MDA-MB-231 parent cells and non-CSCs were not receptive to ATP depletion in the presence of the reported range of the SKC polymer. Even though there was no direct association between the depletion of ATP and SKC1049 cytotoxicity, ATP reduction is one aspect of sensitization of MDRC cells through treatment with Pluronic co-polymers.

Overexpression of BCRP/ABCG2 is also reported in CSCs of breast cancer, pancreatic, hematopoietic, ovarian and others. Results from this study indicated that SKC polymer along with the inhibition of drug-efflux transporters decreased BCRP/ABCG2 expression in CSC cells. Although DXN alone can down-regulate BCRP/ABCG2 expression, its ability was enhanced when combined with SKC polymer. This enhanced activity can be credited to the epigenetic and genetic modulations by Pluronic therapy [212,213].

Cytotoxicity assay verified that SKC1049 was highly effective against CSCs compared to DXN alone, displaying almost the same IC_50_ values as those in non-CSCs. SKC1049 also reduced the colony formation potential of CSCs obtained from both cell lines. In addition to this, SKC1049 demonstrated superior activity in inhibiting tumor growth of orthotropic tumors obtained from both TNBC CSCs when studied in a mouse model in vivo. Lastly, SKC1049 ablated the CSC phenotype in the CSCs obtained tumors. Although, SKC polymers themselves also exhibited tumor growth suppression without DXN and depleting CSCs as well.

In conclusion, the potent action of SKC1049 against oncogenesis in TNBC was demonstrated in vitro and in vivo, via the elimination of drug-resistant CSCs. These discoveries encourage the involvement of Pluronic co-polymers in preventing the occurrence of drug-resistance. Overall, these results signify an easy yet effective therapy strategy to eliminate both, differentiated cancer cells, and small, particularly difficult-to-target drug-resistant CSC populations [172].

CSCs in tumors are believed to be majorly responsible for tumor progression and dissemination. Additionally, they are held accountable for cancer relapse and are highly resistant to present treatment therapies. Thus, CSCs are responsible for therapeutic failures [214]. Circulating tumor cells or CTCs are cells found in the blood of a cancer patient after detaching from the tumor. They detach in the initial stages of cancer and thus are considered the chief markers in fluid biopsies for diagnosis, supervising the therapeutic effect of the drug and monitoring the recurrence of cancer [215].

Gener et al. conducted a study where they employed two fluorescent breast CSC models to be identified via high-throughput screening potential target moieties in CSCs [216]. These models showed red fluorescence (tdTomato) in the presence of a CSC specific promoter, ALDH1A1. This tdTomato red fluorescence is exclusively detected in the CSC sub-population, whereas non-CSCs do not exhibit such a fluorescent marker. This detection segregates non-CSCs from CSC subpopulations and helps in monitoring CSC biological activity before, during and after therapy. Recently, siRNA inhibition of arachidonate 5-lipoxygenase (Alox5) has shown down-regulation its gene expression in vitro and also decrease the cell invasion and malignant transformation of breast CSCs [217].

Zileuton^TM^, an FDA-approved, oral anti-leukotriene drug for treating asthma is a drug inhibitor of ALOX. Its potent anti-cancer activity has been addressed recently during clinical trials. However, a randomized phase II clinical trial that studied the efficacy of Zileuton^TM^ given orally, combined with standard-of-care treatments in cancer patients did not obtain a positive result. The oral route, and hydrophobic nature of Zileuton^TM^, along with high IC_50_ hinders its clinical use in cancer therapies.

To overcome this issue and utilize the potent activity of Zileuton^TM^, Gener et al., decided to formulate Zileuton^TM^-encapsulating polymeric micelles and check the anti-tumor and anti-metastatic activity of these micelles using CSC models in vivo [173]. They used a polymer that is FDA-approved, amphiphilic, flexible in nature, biodegradable and known for its advantages as a drug-delivery system, Pluronic^®^ F127, and loaded Zileuton^TM^ into the Pluronic micelles (PMS-ZN).

As described in their study, due to their stable nature of the micelles and possibility of lyophilization, PMS-ZN are scalable NPs that can be easily produce on a large scale to enable the following translation and clinical applications. Moreover, no toxicity was observed when the maximum feasible dose (MFD) of the drug (15 kg/mg) through micelles was administered into the mice in vivo. The MFD was calculated based on the maximum encapsulation dose and volume that can be administered per mouse. To perform the bio-distribution study of the micelle, they were labeled using a fluorescent dye DIR and injected into a TNBC cell-bearing female athymic mice model. This radio-labeling showed excellent accumulation of PMS-ZN inside the tumor, whereas weak fluorescent signals were detected in the kidney, muscles, plasma and lungs that almost diminished between 24–72 h after administration, signifying clearance of micelles from all these organs. As hypothesized, the CSC sub-population decreased remarkably in both the orthotopic CSC models after treating them with PMS-ZN. Another important observation was the complete abolition of CTCs from the blood stream of mice containing the highly metastatic MDA-MB-231 cell line. As CTCs and mainly CSCs fraction are held responsible for tumor metastatic activity, PMS-ZN thus affects tumor dissemination.

Results confirmed that the metastatic foci present in MDA-MB-231 orthotopic models in vivo were reduced in size after treatment with PMS-ZN, indicating the potential activity of PMS-ZN as an anti-metastatic agent. Further, the combination of PMS-ZN with current anti-cancer treatments could provide synergistic effects and advance present standard-of-care treatments [173].

## 6. Polymeric Nanoparticles for Triple-Negative Breast Cancer Metastasis

Distant metastasis is a very concerning trait of TNBC that often appears in the lung tissues and is more fatal than carcinoma in situ for patients suffering from TNBC [218]. Cancer cells, due to their vigorous proliferation and instant metabolism, exhibit increased amounts of ROS (reactive oxygen species) [219]. Hydrogen peroxide (H_2_O_2_), a by-product produced due to the intra-cellular metabolism of oxygen, is a common and stable ROS and is abundantly found inside tumors [220]. Hydroxyl radicals (OH·), a very toxic ROS is a down-stream product of H_2_O_2_, produced after Fenton reaction catalysis and has the shortest half-life. OH· can quickly demolish bio-macromolecules without dispersing outside the cells and without injuring peripheral cells. It can damage DNA, promote amino acid oxidation and lipid peroxidation. Therefore, OH· could be considered a valuable method for enhancing the effect of tumor treatment by converting abundant H_2_O_2_ present in cancer cells to OH· centered on the Fenton reaction [221].

Besides H_2_O_2_, Fe^2+^ is another chief element of the Fenton reaction. Ferrocene (Frc) can act as an exogenous Fe^2+^ for catalyzing the conversion of H_2_O_2_ to toxic OH· and can help in improving the TNBC therapy strategy [222]. However, hydrophobicity is its main drawback.

Celastrol (Clt), a bioactive compound abundantly found in the roots of *Trypterigiumwilfordii Hook F*., shows great inhibitory action against different sub-types of breast cancer. It is a pentacyclic tri-terpenoid with a partition coefficient of 5.33, making it a poorly water-soluble compound, thus limiting its clinical application. Apart from poor hydrophilicity, its use is also restrained due to its low oral bioavailability, reproductive system-related toxicity and hepato-renal toxicity [223]. Therefore, delivery systems that can resolve all these issues are needed for utilizing Clt against breast cancer/TNBC.

With the advancement in nanotechnology, the bio-reactive elements (such as ROS sensitive, pH-sensitive, enzyme sensitive, etc.) and functional tools (including PEI, PLGA, heparin, etc.) are presented for NPs development. NPs with a pH-sensitive surface have been widely explored as bio-responsive nanocarriers [224]. As the pH in the tumor microenvironment, lysosomes and endosomes is acidic, they act as perfect divisions for site-specific drug release. The β-cyclodextrin and benzimidazole (β-CDT-BZL) complex is a traditional pH-responsive switch that closes at pH 7.4 (neutral environment) and opens in response to an acidic environment, at pH < 6.

Heparin is a biodegradable electro-negative mucoglycan molecule, which is easily degraded in the presence of heparanase (HPN), found abundantly in the tumor microenvironment. Enoxaparin and dalteparin are portions of unfractionated heparin (UF-HR), produced through different degradation procedures and are examples of low molecular weight heparin (LMW-HR). However, LMW-HR has identical pharmacological actions as that of UF-HR. Apart from showing anti-proliferative, anti-metastatic, and anti-angiogenic actions, LMW-HR can successfully bypass first-pass metabolism in the liver leading to the extended blood circulation duration of NPs [225].

Poly-ethylene-imine (PEI), a cationic polymer containing abundant amines at its terminal and is often used for cancer therapy through NPs as it stimulates cellular-uptake and lysosomal escape. Additionally, PEI with low molecular weight is preferred for preparing nano-systems due to its low toxicity [226].

**Figure 4:**
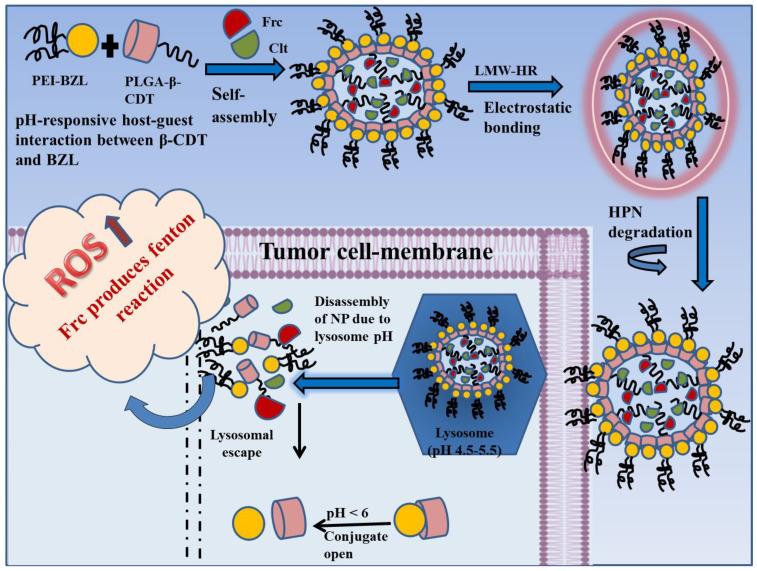
The development and mechanism of action of amphiphilic, pH-sensitive, LMW-HR-coated nanoparticles (PPL-HR NP), containing ferrocene (Frc) celastrol (Clt).

These functional tools have assisted in the design of NPs for quite a long period now. Therefore, Qian and team developed an amphiphilic, pH-sensitive, LMW-HR-coated nanoparticle (PPL-HR NP), containing Frc and Clt. They proposed that PPL-HR NPs will amplify the intra-cellular levels of ROS, enhancing the anti-tumor effect and preventing tumor metastasis because of the presence of LMW-HR. In addition to this the LMW-HR coating would protect the PPL-HR NP from first-pass metabolism, prolonging the persistence of the NPs inside the body [174]. They also proposed the idea of using the pH-responsive molecular switch β-CDT-BZL for linking PEI-BZL and PLGA-β-CDT to produce NPs (PPL NP) which could undergo self-assembly at physiological pH~7.4 and then dis-assemble inside lysosomes at pH < 6, releasing Fcr and Clt (Figure 4). Upon release inside the tumor, Clt induces apoptosis, promoting tumor-cell killing and Fcr stimulates ROS production, strengthening and promoting the therapeutic effects of Clt.

The % DL (drug loading) of Fcr and Clt in PPL-HR NPs was found to be 2.83% and 3.08%, respectively and the % EE was 90.73% for Fcr and 98.56% for Clt. High % DL and % EE were credited to the hydrophobic nature of Fcr and Clt, which promoted loading of both drugs inside the hydrophobic PLGA core of the NPs.

To verifying the action of Fcr, crystal violet (CV) was used, and solutions of CV, CV+H_2_O_2_ and CV+H_2_O_2_+Fcr were prepared using different concentrations. The solutions were kept at room temperature for 90 min and then their absorbances were measured at 580nm using a UV-spectrophotometer. The absorbance value of CV reduced after the addition of Fcr, indicating that a Fenton reaction occurred and Fcr had converted H_2_O_2_ to HO·.

Tumor cells have a high level of H_2_O_2_; thus, a steady source of providing H_2_O_2_ for Fenton reactions. The beneficial conversion ability of Fcr guarantees H_2_O_2_ catalysis into HO·, thus synergizing with the tumor-killing ability of Clt.

Further, to check the in vitro release of Fcr and Clt due to the breaking of the β-CDT-BZL bond at low intra-cellular pH, a release study was performed at different pH. At pH 7.4, the pH of blood and normal tissues, the cumulative release of Fcr from PPL-HR NP was about 31.24 ± 2.47% at the 48th hour and it reached 85.89 ± 2.71% in 48 h at pH 5.8, i.e., the pH of endosomes. Up to 80.23 ± 3.49% Clt was released in 48 h at pH 5.8, while only 23.58 ± 3.81% was released at pH 7.4 at the same time point. This rapid release of the drug at low pH proves the pH-sensitive behavior of the NPs, which is advantageous for enhanced the intra-cellular release and improved cytotoxicity.

**Figure 5:**
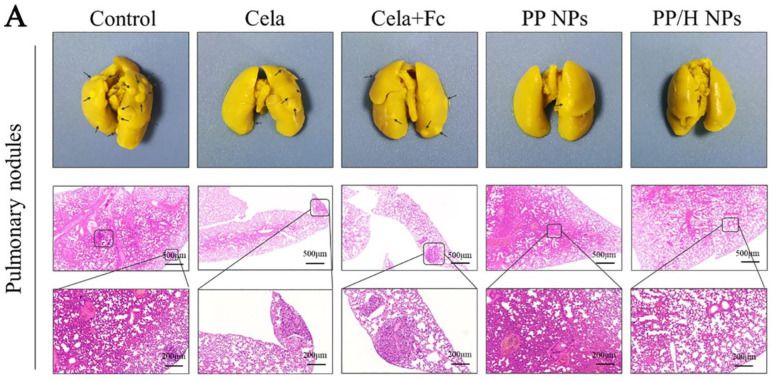
Observation of pulmonary metastasis after administration of different formulations analyzed by H&E staining. A looser tissue was observed in mice lung treated with PP/H compared to the others with no dense staining. Reproduced with permission from reference [174].

Further, an in vivo study was done using female Balb/c mice and for studying the anti-metastatic action of the developed NPs, the lung tissues of mice were analyzed. It was observed that pulmonary-metastatic nodules were present at the periphery of the lungs and spherical protrusions of different sizes were present. The control group showed most nodules reflecting prominent lung metastasis. In the free drug group, the nodes were reduced significantly, compared to the control group, indicating the anti-metastatic potential of Clt in TNBC treatment. Further, PPL-HR NP displayed the most observable anti-metastatic result, and the nodules were rarely present at the lungs periphery of this group. The lung tissues treated with the PPL-HR NPs were considerably looser compared to other groups, and there were no deep dense staining areas observed when stained with hematoxylin and eosin (H&E) (Figure 5). The enhanced cytotoxic effect of PPL-NP along with the anti-metastatic activity of LMW-HR enhanced the overall anti-tumor action of the developed NPs [174].

Targeting multiple features to relieve the aggressive nature of TNBC has gained great importance in TNBC therapy. Angiogenesis and cellular migration are two hallmark features of metastatic TNBC. Angiogenesis refers to the development of new blood vessels from already existing vessels that nurture the tumor by providing required nutrients and preparing a getaway route for the tumor cells to escape and enter the blood circulation. To stimulate the angiogenic phenotype, angiogenic factors such as VEGF-A are released from proliferating tumor cells [227]. Besides regulating angiogenesis, VEGF-A also exerts an autocrine effect that influences the migration of cancer cells directly which is an essential process in cancer metastasis [228].

Tumor cells can move either individually or together in a group. When migrating individually, they move through two different morphologies. One is a mesenchymal morphology the other is an amoeboid morphology. The mechanism behind these morphologies of cell migration is regulated by the Rac1/RhoA regulatory circuit [229]. The mesenchymal mode is regulated mainly by Rac1 signaling while the amoeboid mode depends heavily on RhoA/ROCK 1 signaling. Therefore, TNBC therapy that can manipulate VEGF-A, RohA, and Rac1 collectively to restrict both, angiogenesis and cell migration for an improved prognosis of TNBC can change the fate of TNBC treatment [230].

Thymoquinone (TQN), is a potent phyto-compound and active constituent of *Nigella sativa* (black seed) extracted essential oil. The anti-cancer activity of TQN has been extensively studied and it has been found that TQN can regulate a series of signaling pathways involved in cancer progression. However, the anti-cancer efficacy of TQN is obstructed because of its hydrophobic nature, instability at high temperatures and instant deterioration in aqueous media all reducing the bioavailability of potent TQN [231].

To circumvent such concerns, Bhattacharya and his colleagues in their research on TNBC treatment produced polymeric NPs of TQN using amphiphilic, biodegradable Pluronic^®^ block co-polymers and coated them externally using HA (TQN-P-HA) [175]. Pluronic^®^ co-polymer is composed of one lipophilic region of PPO (poly-propylene oxide) and two hydrophilic PEO (poly-ethylene oxide) arranged in a triblock structure “PEO-PPO-PEO”. In an aqueous medium, when at or beyond the critical micelle concentration (CMC), Pluronic^®^ co-polymers show self-assembly to form polymeric micelles of nano size (generally less than 100nm), thus efficiently entrapping the hydrophobic drugs inside.

**Figure 6:**
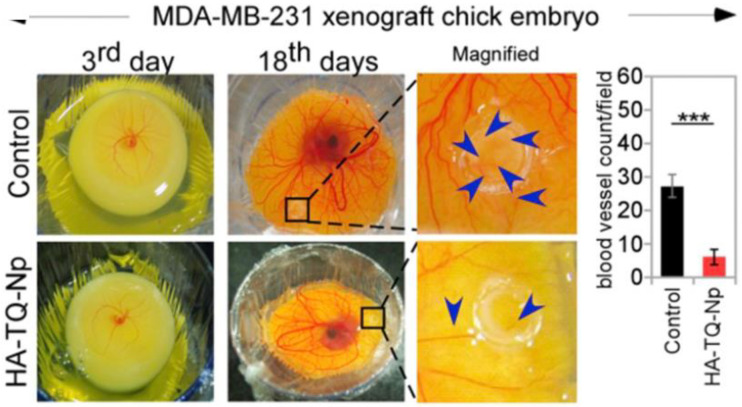
Digital images of TQN-P-HA NP-untreated (control) and -treated MDA-MB-231 chick embryo xenograft models showing the angiogenic activity in both the groups. Reproduced with permission from reference [175]. *** *p* < 0.001.

The results of the mechanistic study highlighted that TQN-P-HA NPs regulate TNBC cell migration by up-regulating microRNA-361 that down-regulates Rac1/RhoA regulatory circuit-facilitated cell migration. The NPs agitate cell migration through the autocrine effect of VEGF-A. Furthermore, the down-regulation of VEGF-A by TQN-P-HA NPs also hinders tumor-induced vascularization. The anti-angiogenic and anti-metastatic action of TQN-P-HA NPs was verified in both the 4T1-mammary tumor mice model and MDA-MB-231 chick embryo xenograft model. In the chick embryo model, it was found that in the control group plenty of blood vessels were seen in the tumor, while in the TQN-P-HA NP-treated group showed very few blood vessels (Figure 6). Additionally, in the TQN-P-HA NPs xenograft embryo model, very few human cells metastasized to the lungs and liver of the embryos. All these results, therefore, vouch that this innovative tumor-targeted approach can thus inhibit angiogenesis and metastasis simultaneously, relieving the tumor burden and resulting in better management of TNBC [175].

Folate receptors (FRs) are overexpressed in various types of cancers including TNBC; however, they are usually not expressed or expressed at very low levels in healthy cells, making these receptors an interesting target for cancer therapy [232].

Given that, antibodies and many different ligands used for cancer-cell targeting after internalization are transported to lysosomes where they are destroyed. Since folic acid (FA) is a necessary vitamin for performing specific cell functions, when customize as a targeting ligand, the carrier FA is detached inside an endocytic vesicle or released inside the cytoplasm, offering the benefit of its use [233].

C’e and colleagues in a study developed FA-conjugated DXN-loaded lecithin-polysorbate-80 chitosan-coated lipid core nanocapsules (FA-DXN-LPC-L-NCs). By simultaneously using two different ligands for the first time to produce organo-metallic complexes, they demonstrated the possibility of constructing lipid core-dual functionalized nanocapsules. They elucidated the chemical structure via physiochemical characterization, and the function of lecithin in this supramolecular assembly was recognized, which interacts with the chitosan and polymer wall, concurrently [234].

As it is already established, that TNBC cells overexpress folate receptors, they further determined the efficacy of FA-DXN-LPC-L-NCs in MDA-MB-231 cells in vitro. Cell viability assays, migration assays, quantitative real-time PCR (qRT-PCR), nitric oxide (NO) production analysis, cellular uptake studies and the clonogenic assays were performed.

Different gene expressions were evaluated by qRT-PCR and upon treatment with FA-DXN-LPC-L-NCs, an increased expression of *Cat* and *Mnsod* genes compared to the free DXN-treated group was observed [176]. The *Cat* gene is a protective manager, protecting cells from the harmful effects of hydrogen peroxide. The expression of this gene is mediated by several factors such as PPAR γ (peroxisome proliferator-activated receptor γ), TNF-α (tumor necrosis factor-alpha), etc. *MnSod*, a member of the manganese-superoxide dismutase/mitochondrial iron family, eliminates ROS generated through oxidative phosphorylation. *MnSod* also acts as a major protective agent against oxidative stress and cell injury due to inflammation [235].

The migration capability of MDA-MB-231 cells was studied after treatment with the FA-DXN-LPC-L-NCs. Cell migration is a feature of cell metastasis. After 6 h of treatment, significant inhibition in the migration of cells was observed. The width of the scratch was lessened by about 43% after 6 h of incubation and to 90% after 9 h. The results signify that FA-DXN-LPC-L-NCs remarkably reduce cancer cell migration in vitro [176].

Nitric oxide/NO is a crucial component of cell signaling. NO liberated after i-NOS activation has cytostatic and cytotoxic effects, which in-turn promotes the destruction of parasites, microorganisms and sometimes tumor cells. Additionally, NO cytotoxicity can induce inflammation. After treatment with the developed formulation, the levels of NO dramatically increased. However, there was negligible increase in NO levels after treating MDA-MB-231 cells with free DXN. The NO expression was 7.6 µmol after FA-DXN-LPC-L-NC treatment and only 5.8 µmol after treatment with free DXN. This observation indicated an oxidative stress-modulating activity of the developed FA-DXN-LPC-L-NCs. All these data together signify the strong effective activity of FA-DXN-LPC-L-NCs against the MDA-MB-231 TNBC cell line [176].

Photodynamic therapy (PDT) is a budding non-invasive cancer therapy that employs photosensitizers (PSZs) for the killing of cancer cells. These PSZs are activated by absorbing light and then release enormous amounts of singlet oxygen (O_2_^1^) [236].

PDT offers various advantages such as greater selectivity, low risk of injury, minimum systemic toxicity, and good effects compared to conventional treatments. However, its clinical application is limited due to the poor penetrative ability of visible light applied to human tissues and the low bioavailability of PSZs. Using lanthanide-doped up-conversion NPs (LUCVNPs) that can be activated through tissue-penetrating NIR (near-infrared) light and then emit UV (ultraviolet or visible) light could emerge as a potential solution to the given problems. Therefore, under NIR, PSZs bonded to LUCVNPs are able to produce a huge quantity of cytotoxic O_2_^1^ as LUCVNPs can convert NIR into visible light, which can then be absorbed by PSZs which leads to deep tissue therapy.

Apart from this, Rose Bengal (RBL) can be covalently bonded with LUCVNPs which will contribute to overcoming the low tumor penetrating ability and fast clearance of conventional PDT. Therefore, in order to attain the enhanced synergistic anti-cancer effect of chemotherapeutic and PDT, Jin and co-workers conjugated DXN with LUCVNPs using thioketal (TL), a sulfhydryl-dependent ROS-responsive linker that can be immediately demolished by O_2_^1^ [177]. Even though NP accumulation at the tumor site is regulated by the EPR effect, their distribution is restricted in metastatic lesions as well as in macrophage phagocytosis. Recent studies have reported the presence of particular membrane proteins, for example, CD47, and EpCAM on the cancer cell membranes that regulate immune tolerance and the cell adhesion ability of cancer cells to avoid macrophage uptake [237]. Therefore, the team decided to camouflage LUCVNPs using an outer layer of the cancer cell membrane (CM), so that the NPs could bio-mimic cancer cells and show the immune escaping and tumor-targeting characteristics of cancer cells. They prepared a DXN-entrapped, ROS-sensitive-PEG polymer and an RB-integrated-LUCVNP co-delivery structure (DXN-TPR-LUCVNPs), and further modified it using cancer CMs (DXN-TPR-LUCVNPs/CM). Once the DXN-TPR-LUCVNPs/CM reaches the tumor site, LUCVNPs is activated using an NIR laser, causing the PDT process to occur. Furthermore, high ROS demolish the ROS-sensitive linker, promoting self-degradation of the DXN-TL-PEG polymer on the LUCVNPs, leading to the instant release of DXN into the tumor site to facilitate chemo-photodynamic synergistic combinatorial therapy [177]. Combining PDT and chemotherapy can destroy solid cancer cells dramatically via necrosis and apoptosis. Furthermore, cancer cell destruction through PDT provokes a host-immune response to eliminate metastatic tumors, by promoting the release of cancer cell debris, which further causes immunogenic cell death (ICD). However, this response imitated by ICD is greatly restricted in vivo because of the extensive immune-suppression due to the adenosine pathway. As cancer cells experience ICD, extracellular ATP (adenosine triphosphate) is released quickly. However, excessive ATP is hydrolyzed into adenosine through CD73, a potential immune-suppressing ectoenzyme that acts as a negative mechanism to avert a T cell immune response. This CD73 is found to be overexpressed on breast tumor cells leading to a poor prognosis. Recent research found that anti-CD73 antibodies alone showed no effect but when combined with radiotherapy or chemotherapy, an immune-checkpoint blockade leads to a synergetic anti-tumor response and anti-metastatic activity [238]. Therefore, to achieve the maximum anti-cancer effect, Jin and co-workers further combined chemotherapy, PDT and CD73 blockade.

In conclusion, potent cancer CM-concealed, multifunctional LUCVNPs were developed. The PSZs-RB and ROS-activated polymer PEG-DXN-TL were effectively complexed with LUCVNPs and cancer CMs were further used to coat the NPs. The CM-concealed LUCVNPs aided both their innate targeting capability and immune escape ability. The in vivo results also confirmed the combinatorial action of DXN-TPR-LUCVNPs/CM and CD73 blockade with an excellent anti-progression effect on the tumor without any systemic adverse effects. The impulsive anti-cancer immunity was intensified by the combination activity of chemotherapeutic DXN, PDT-activated ICD and blocking of CD73. Therefore, this novel bio-mimicking multi-functional fusion of the LUCVNPs with anti-CD73 antibodies could prove to be a promising regimen for targeting and treating metastatic TNBC [177].

In breast cancer cells, levels of copper (Cu) are often elevated, stimulating cell proliferation, migration and cancer progression. Thus, inhibiting this copper level enhancement can in turn inhibit endothelial cancer cell motility and proliferation and hinder tumor angiogenesis in vivo [239]. A well-known copper chelator, tetrathiomolybdate (TTM), has a powerful suppressing activity against tumor growth and angiogenesis as TTM induces copper deficiency. TTM is currently under clinical trials. However, there are some unwanted side effects of TTM such as emesis, optic neuritis, leukopenia, and erythra. To use this anti-cancer property of Cu chelators, Zhou and his co-workers developed a pH-sensitive polymeric Cu chelator with a tumor-cell targeting property and utilize it to formulate NPs for the cancer cell-targeted delivery of resiquimod (R848) [178].

**Figure 7:**
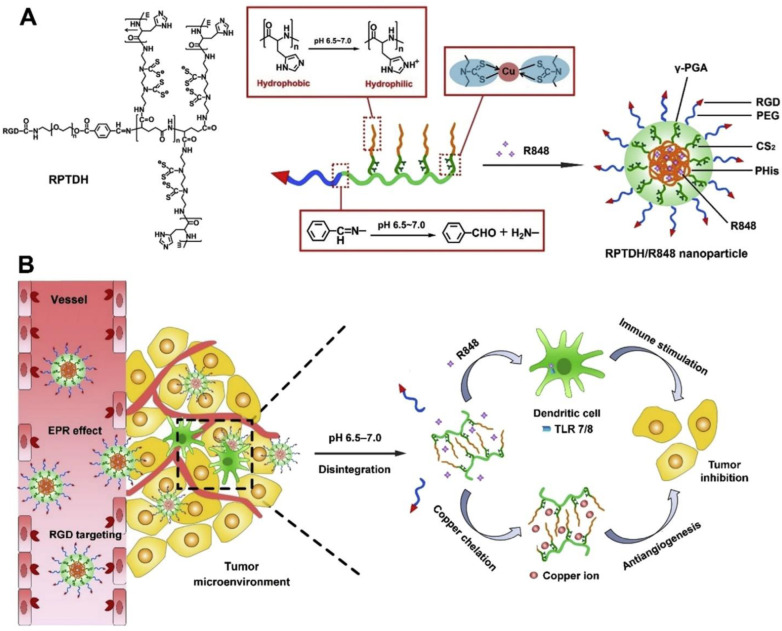
Illustration of the (**A**) preparation of the RGPPTDHist-R848 NPs and (**B**) its mechanism of action in breast cancer cells [178].

R848 is a TLR (toll-like receptor)-7/8 agonist with a potential anti-cancer effect as it activates dendritic cells (DCs). The formulated NPs allowed efficient treatment of breast cancer by combining immune activation and copper chelation along with the controlled release of R848 [178]. To formulate the co-polymer, they utilized a specific Cu chelator, triethylenetetramine-bis (dithiocarbamate) (TETA-DTC) [240], and grafted it onto poly-gamma-glutamic acid (γ-PGA) through amide bonding. Then, poly-L-histidine (PL-Hist), a pH-responsive polymer, was attached to the TETA-DTC through its end amino group. RGD (Arg-Gly-Asp) peptide was conjugated with PEG and further introduced to γ-PGA through acid-sensitive benzoic-imine bonding to form a coil–comb block polymer RGD/PEG-b-γ-PGA-g-(TETA-DTC-PL-Hist) (RGPPTDHist). At weak alkaline and neutral pH, RGPPTDHist self-assembles to form NPs because of aggregation of PL-Hist due to hydrophobic interactions and thus hydrophobic R848 can be loaded into it. After intravenous administration, NPs were found to be stable at normal physiological pH. They attached to the targeted cancer cell through binding between the overexpressed tumor cell integrin αvβ3 and RGD peptide and were uptaken by cells through the EPR effect. As the tumor microenvironment is weakly acidic, these NPs become destabilized due to benzoic-imine bond cleavage and lipophilic/hydrophilic transition of PL-Hist and finally disintegrates, thus gradually releasing R848 leading to DC activation via the TLR-7/8-mediated signaling pathway. The remaining polymer, γ-PGA-g-(TETA-DTC-PL-Hist) additionally exerted an anti-cancer and anti-angiogenic effect via copper chelation. RGPPTDHist-R848 NPs constrained the cell movement, invasion and vascular tube formation in HUVECs (human umbilical vein endothelial cell) cells line through copper chelation, in vitro, signifying their remarkable anti-angiogenic action. Moreover, RGPPTDHist-R848 NPs significantly promoted activation and maturation of human plasma-cytoid dendritic CAL-1 cells, demonstrating the immune-activation activity of the NPs. When studied in vivo with mice bearing different cell lines, including MDA-MB-231, 4T1, MCF-7 breast cancer cell lines and BEAS-2B (normal lung epithelial cell line), the RGPPTDHist-R848 NPs exhibited tremendous tumor-targeting capability for both primary and secondary breast tumors and lung metastases. Thus, dramatic tumor growth suppression and anti-metastatic activity can be seen through Cu deficiency-induced anti-angiogenesis and R848-activated immune response [178] (Figure 7).

## 7. Conclusions, Current Challenges and Prospects

Breast cancer, specifically TNBC is becoming the center of current research because of the lack of adequate TNBC therapies compared with other hormone-positive breast cancers. Researchers have revealed that profound knowledge of TNBC pathology is the chief element for developing better functioning NPs. Tumor target-based approaches and regulated release of the payload is considered a pre-requisite for targeting any type of cancer. Owing to their physiochemical properties and multifunctional nature, polymeric nanocarriers shine out as a promising method for TNBC treatment. Targeting molecules carried by these NPs are designed specifically to intermingle with targets such as αVβ3 integrins, neuropilins, somatostatin receptor 2, etc., which are found to be highly overexpressed on the TNBC cell surface. Along with this, the modifiable structure of the polymers to prepare stimuli-responsive NPs results in a more site-specific and controlled release of therapeutic agents. Many researchers have investigated polymeric NPs as polymers are biocompatible, biodegradable, offer improved stability, solubility, bioavailability and allow for a modifiable drug/cargo release pattern which can synergistically be applied for delivering cargo into the tumor microenvironment. Surface-modified and non-modified polymeric NPs were discussed in this article. Many NPs have been effective in targeting certain receptors overexpressed in TNBC cell lines, in vitro and animal xenograft in vivo models,. They exhibit excellent biocompatibility, high transfection efficacy, enhanced cellular uptake and low toxicity. Even though there are proven advantages of polymeric NPs, their commercialization is still challenging. Their formulation has been limited to academic labs only and their clinical transformation seems to be a dubious dream. Most of the prepared polymeric NPs have been administered via the parental route, which is an invasive procedure leading to patient noncompliance. The scaling-up process is accompanied by a high-cost of production and low production yield. Furthermore, the clinical toxicity of some polymers is another serious factor. Toxicity is affected majorly by the concentration of polymers/co-polymers and their degree of polymerization. It also depends on the size, structure, and coating of the NPs. Therefore, optimizing the molecular features of the polymers/co-polymers is a crucial step for minimizing polymeric toxicity. The immunological response to polymeric NPs should also be taken into consideration due to the already established anti-PEG immune response. Designing and using hydrophilic polymers to oppress such immunogenicity should be given attention. However, polymeric nanocarriers still fail to progress in clinical trial due to stability-related issues. Moreover, the use polymeric nanocarriers and tumor suppressing genes in pre-clinical studies is extremely low indicating its redundancy in clinical settings. Hence, more pre-clinical and clinical studies are needed to be performed with pronounced modification to amplify the existing results and boost the oncological therapy. Considering and correcting such challenges will help in designing better polymeric NPs in the future. Nonetheless, chemotherapeutic-loaded polymeric NPs is an outstanding approach to transport cargo directly into tumor cells, thus providing an effective treatment of TNBC.

## Figures and Tables

**Table 1:** Research work conducted on the tumor targeting ability of different polymeric nanocarriers in complex with different chemotherapeutic moieties.

Therapeutic Moiety	Polymer Used	Additional Chemical Moiety	Final Preparation	Type of Study	Cell Line Used	Animals Used	Outcomes Obtained	Ref
POLYMERIC NANOPARTICLES FOR THE TREATMENT OF TRIPLE-NEGATIVE BREAST CANCER								
Paclitaxel (PTX) and miRNAi-221/222	PEG-PLGA	Calcium phosphate	miRNAi-221/222 encapsulated in calcium phosphate, PTX encapsulated in DOPA, both further encapsulated in PEG-PLGA nanoparticles	In vitro	MDA-MB-231 cell line	-	Synergistic action of combining a conventional chemotherapeutic drug (PTX) with RNA interference (miRNAi-221/222) was achieved. This combination helped in reducing the dose of potent cytotoxic drugs without compromising its cytotoxicity.	[107]
Doxorubicin (DXN), Erlotinib (ETB)	PLA-b-PEG		PLA-b-PEG nanoparticle	In vitro and in vivo	MDA-MB-231 cell line	R7 cell line containing FBV female mice	Formulated polymeric nanoparticles co-localized inside the tumor, showed improved therapeutic efficacy and minimum systemic toxicity by differentiating tumor tissues from the healthy ones	[109]
Doxorubicin (DXN),	poly(N-isopropylacrylamide)	-	poly(N-isopropylacrylamide)-coated DXN-loaded tadpole-shaped nanostructure	In vitro	MDA-MB-231 cell line	-	Tadpole shape exhibited about 15 times increase in NPs cellular uptake in comparison to spherical-shaped NPs composed of same polymer.	[112].
Embelin (EBN) and TRAIL plasmid	PEI and PBAE	Hyaluronic acid (HA)	HA-coated PEI and PBAE nanoparticles (HA-PPB NPs)	In vitro	CD44 over-expressing MDA-MB-231	-	HA-PPB NPs acted as a potent carrier for co-transporting therapeutic genes along with anti-cancer drugs. Combination of pTRAIL and EBN can be used as a potent synergistic therapy for TNBC treatment.	[117]
Piperine (PPN)	PEI–PLGA		PPN-loaded PLGA–mPEG co-polymers nanoparticle (PPP-NPs)	In vitro	BT-549 and MDA-MB-468 cell line	-	Polymeric nanoparticles helped in delivering hydrophobic PPN without comprising with its cytotoxicity	[130]
Doxorubicin (DXN)	HPMA and mPEG	-	DXN -encapsulating HPMA-b-methoxy PEG co-block polymeric micelles	In vitro and in vivo	4T1 murine and MDA-MB 231 and MCF-7 cell line	Female Wistar rats	Micelles formed using polymer with HPMA:mPEG and drug:polymer ratio of 175:1 and 1:10, respectively resulted in nanoparticles with particle size distribution of narrow range and highest drug loading, compared to other synthesized ranges of polymer.	[135]
Doxorubicin (DXN) and Paclitaxel (PTX)	(P MEO_2_MA-co-OEGMA-co-DMAEMA-b-PLGA)	Poly-dopamine (PDA)	DXN and PTX encapsulated encapsulating (P MEO_2_MA-co-OEGMA-co-DMAEMA-b-PLGA) co-polymer nanoparticles, surface modified with PDA	In vitro and in vivo	MDA-MB-231 tumor cell line	Female Balb/C mice	Surface-modified PDA prevented burst release of drug from the nanocomposite. Nanoparticles produced sufficient heat required for photothermal therapy, under localized NIR irradiation and thus precise thermo-responsive drug release was achieved.	[143]
Docetaxel (DCL)	Poly(ethyleneglycol)-b-poly (lactide)-co-poly(N3-alpha-ε-caprolactone)		Docetaxel-loaded micellar-like nanoparticles (MR-NPs)	In vitro	TNBC cell line (MDA-MB-231), breast cancer cell line (MCF7) and normal breast cell line (MCF10A)	-	Docetaxel-loaded MR-NPs with reducible cross-links exhibited greater efficacy in 2D, 3D in vitro TNBC models by acting against the abnormal cell biology of TNBC.	[144]
Doxorubicin (DXN)	PLA-diazobenzene-PEG di-block co-polymer	iRGD peptide	DXN encapsulating self-assembled hypoxia-sensitive polymersomes (PMs), surface-conjugated with iRGD	In vitro and in vivo	MDA-MB-231 cell line	Female nude mice	Significant enhancement in drug release from the polymersomes in the hypoxic environment was observed	[148]
Cholesteryl biguanide-conjugated hydrochloride (CBCH) and Magnolol (MGL)	mPEG-PLGA	Aminoethyl anisamide ligand	AEAD-PEG-PLGA conjugated mPEG–PLGA-coated CBCH and MGL nanomicelles	In vitro and in vivo	Murine 4T1 TNBC cell line	Female Balb/c mouse model	An effective NP system for TNBC treatment was formulated	[162]
Metformin	PEG–PLGA	Hyaluronic acid (HA) nanoparticles	Metformin-encapsulated graphene oxide NPs	In vitro and in vivo	Murine 4T1 TNBC cell line	Female Balb/c mouse model	Novel graphene oxide nanoparticles successfully encapsulated metformin and exerted anti-cancer activity against TNBC cell line	[164]
**POLYMERIC NANOPARTICLES FOR TRIPLE-NEGATIVE BREAST CANCER IMMUNOTHERAPY**								
Polyinosinic–polycytidylic acid 17[Poly (I–C)]	PVA and PEI chlorin e6 (PEI-C-e6)		NIR light-regulated charge-reversal [Poly(I–C)] nanoparticles (NCRNPs-[Poly(I–C)])	In vitro and in vivo	Murine 4T1 TNBC cell line	Female Balb/c mouse model	The NCRNPs-[Poly(I–C)] provide a promising strategy for the controlled release of nucleic acid-based immunomodulators that may improve the photodynamic cancer immunotherapy of TNBC	[170]
CD155 siRNA and PD-L1 antibodies	mPEG-PLL-PLGA (PPGPL)		PPGPL-_CD155si_/P nanoparticles	In vitro and in vivo	Murine 4T1 cell line	4T1 tumor bearing Female Balb/c mice	A potent combination approach for immunotherapy treating PD-L1/CD155+ TNBC. This formulation can be widely applied for treating CD155 and PD-L1 co-expressing cancers.	[171]
**POLYMERIC NANOPARTICLES FOR THE TREATMENT OF CANCER STEM-CELLS IN TRIPLE-NEGATIVE BREAST CANCER**								
Doxorubicin (DXN).	Pluronic F127 and L61,		Doxorubicin-loaded Pluronic F127, L61 polymeric micelles		Basal MDA-MB-468 and claudin-low MDA-MB-231 TNBC cell lines	Female athymic mice	These discoveries encourage the involvement of Pluronic co-polymers in preventing the occurrence of drug-resistance.	[172]
Zileuton	Pluronic^®^ F127	-	Zileuton–Pluronic^®^ F127 polymeric micelles	In vitro and in vivo	MDA-MB-231 tumor cell line	MDA-MB-231 tumor cell-bearing female athymic mice	Remarkable intra-tumoral reduction in CSC, reduction in CTCs and CSCs in the blood stream of tumor-bearing animal models	[173]
**POLYMERIC NANOPARTICLES FOR THE TREATMENT OF TRIPLE-NEGATIVE BREAST CANCER METASTASIS**								
Ferrocene (Frc) and Celastrol (Clt),	PEI-PLGA	Low molecular weight heparin (LMW-HR) for anti-metastatic activity	Amphiphilic, pH-sensitive, LMW-HR-coated nanoparticle	In vitro and in vivo	Murine 4T1 cell line	3T3/4T1 tumor-bearing Female Balb/c mice	The cytotoxic effect of polymeric nanoparticles and anti-metastatic activity of LMW-HR enhanced the overall anti-tumor action of the developed nanoparticles.	[174]
Thymoquinone (TQN)	Pluronic block co-polymer	Hyaluronic acid (HA)	HA-coated TQN loaded, Pluronic nanoparticles (TQN-P-HA NPs)	In vitro and in vivo	4T1 and MDA-MB-231	4T1-mammary tumor mice model and MDA-MB-231 chick embryos xenograft model	In TQN-P-HA NP-treated group, very few blood vessels were seen in the xenograft model, indicating anti-angiogenesis activity and in the TQN-P-HA NP-treated xenograft embryos models, very few human cells were metastasize to the lungs and liver of the embryos, signifying an anti-metastatic effect.	[175]
Doxorubicin (DXN),	Chitosan	Lecithin	Folate receptor-conjugated doxorubicin-loaded lecithin-polysorbate 80-chitosan-coated lipid core nanocapsules (FA-DXN-LPC-L-NCs)	In vitro	MDA-MB-231 cell line	-	Results obtained after different in vitro studies including cellular uptake assay, oxidative stress assay, gene expression evaluation, and migration assay revealed the promising activity of FA-DXN-LPC-L-NCs against TNBC.	[176]
Doxorubicin (DXN) and anti-CD73 antibody	PEG	Rose Bengal, Thioketal	lanthanide-doped up-conversion nanoparticles (LUCVNPs)	In vitro and in vivo	Murine 4T1 cell line	Balb/C mice	Novel bio-mimicking multi-functional fusion of LUCVNPs with anti-CD73 antibodies could prove to be a promising regimen for targeting and treating metastatic TNBC	[177]
Resiquimod (R848)	poly-L-histidine (PL-Hist)	Triethylenetetramine-bis (dithiocarbamate) (TETA-DTC), RGD (Arg-Gly-Asp) peptide	Resiquimod loaded-(TETA-DTC), RGD-(PL-Hist) nanoparticles	In vitro and in vivo	Human MDA-MB-231, Murine 4T1, MCF-7 breast cancer cell lines and BEAS-2B (normal lung epithelial cell line)	Female Balb/C mice	Remarkable tumor growth suppression and anti-metastasis activity via Cu deficiency-induced anti-angiogenesis and R848 activated immune response	[178]
